# An alternative route for β-hydroxybutyrate metabolism supports cytosolic acetyl-CoA synthesis in cancer cells

**DOI:** 10.1038/s42255-025-01366-y

**Published:** 2025-09-08

**Authors:** Faith C. Kaluba, Thomas J. Rogers, Yu-Jin Jeong, Rachel ‘Rae’ J. House, Althea Waldhart, Kelly H. Sokol, Samuel R. Daniels, Cameron J. Lee, Joseph Longo, Amy Johnson, Vincent J. Sartori, Ryan D. Sheldon, Russell G. Jones, Evan C. Lien

**Affiliations:** 1 Department of Metabolism and Nutritional Programming, Van Andel Institute, Grand Rapids, MI USA; 2 Van Andel Institute Graduate School, Grand Rapids, MI USA; 3 Mass Spectrometry Core, Van Andel Institute, Grand Rapids, MI USA

**Keywords:** Cancer metabolism, Cancer microenvironment, Metabolism, Lipids

## Abstract

Cancer cells are exposed to diverse metabolites in the tumour microenvironment that are used to support the synthesis of nucleotides, amino acids and lipids needed for rapid cell proliferation. In some tumours, ketone bodies such as β-hydroxybutyrate (β-OHB), which are elevated in circulation under fasting conditions or low glycemic diets, can serve as an alternative fuel that is metabolized in the mitochondria to provide acetyl-CoA for the tricarboxylic acid (TCA) cycle. Here we identify a non-canonical route for β-OHB metabolism that bypasses the TCA cycle to generate cytosolic acetyl-CoA. We show that in cancer cells that can metabolize ketones, β-OHB-derived acetoacetate in the mitochondria can be shunted into the cytosol, where acetoacetyl-CoA synthetase (AACS) and thiolase convert it into cytosolic acetyl-CoA. This alternative metabolic routing allows β-OHB to avoid oxidation in the mitochondria and to be used as a major source of cytosolic acetyl-CoA, even when other key cytosolic acetyl-CoA precursors such as glucose are available in excess. Finally, we demonstrate that ketone body metabolism, including this alternative AACS-dependent route, can support the growth of mouse *Kras*^G12D^; *Trp53*^−/−^ pancreatic tumours grown orthotopically in the pancreas of male mice, as well as the growth of mouse B16 melanoma tumours in male mice fed a calorie-restricted diet. Together, these data reveal how cancer cells use β-OHB as a major source of cytosolic acetyl-CoA to support cell proliferation and tumour growth.

## Abstract (web summary)

Kaluba, Rogers et al. show that in cancer cells that can metabolize ketones, they may reroute them to produce cytosolic acetyl-CoA and support tumour growth.

## Main

**Fig. 1:**
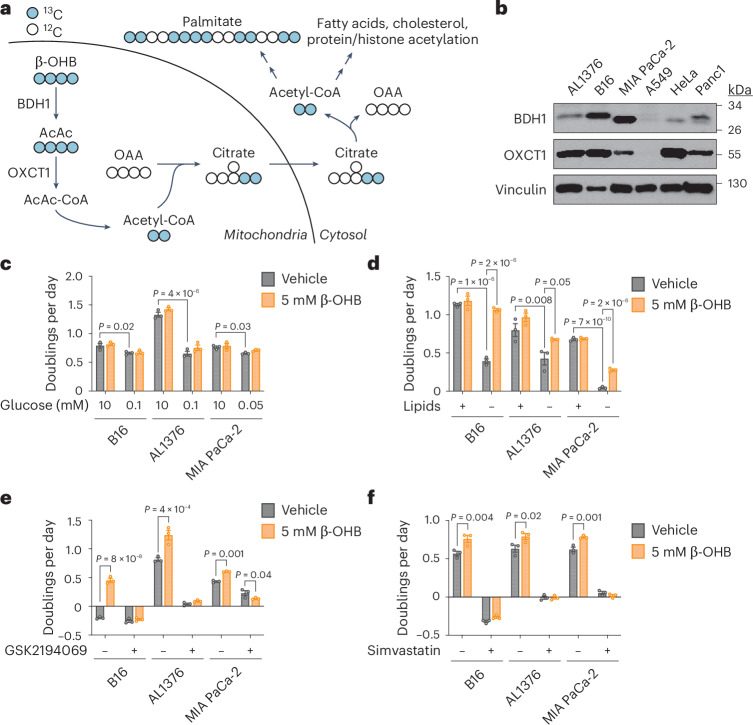
β-OHB promotes the proliferation of lipid-starved cancer cells. **a**, Schematic of β-OHB metabolism and ^13^C labelling derived from [U-^13^C]-β-OHB. AcAc, acetoacetate; OAA, oxaloacetate. **b**, Immunoblot for BDH1, OXCT1 and vinculin in the indicated cancer cell lines. **c**, Proliferation rates of the indicated cancer cell lines grown in high or low glucose conditions, with or without 5 mM β-OHB. **d**, Proliferation rates of the indicated cancer cell lines grown in lipid-replete versus lipid-depleted culture media, with or without 5 mM β-OHB. **e**,**f**, Proliferation rates of the indicated cancer cell lines grown in lipid-depleted media, with or without 5 mM β-OHB and 0.3 µM of the FASN inhibitor GSK2194069 (**e**) or 25 µM of the cholesterol synthesis inhibitor simvastatin (**f**). Data are presented as means; error bars, s.e.m.; *n* = 3 biologically independent replicates. Comparisons were made using a two-way ANOVA (**c**–**f**). Source data

**Extended Data Fig. 1:**
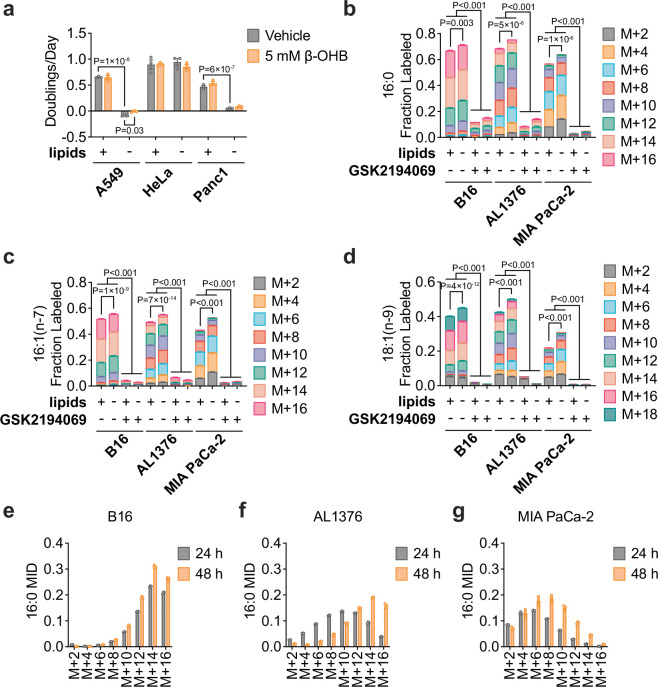
β-OHB is used for fatty acid synthesis. Related to Fig. 1. **a**, Proliferation rates of the indicated cancer cell lines grown in lipid-replete versus lipid-depleted culture media, with or without 5 mM β-OHB. **b-d**, Palmitate (16:0) mass isotopomer distribution (MID) (**b**), palmitoleate (16:1(n-7)) MID (**c**), and oleate (18:1(n-9)) MID (**d**) in B16, AL1376, and MIA PaCa-2 cells labeled with 5 mM [U-^13^C]-β-OHB for 24 h in lipid-replete versus lipid-depleted culture media, treated with or without 0.3 µM of the FASN inhibitor GSK2194069. **e-g**, 16:0 MID in B16 (**e**), AL1376 (**f**), and MIA PaCa-2 (**g**) cells labeled with 5 mM [U-^13^C]-β-OHB for 24 or 48 h in lipid-replete culture media. Data are presented as mean ± s.e.m; *n* = 3 biologically independent replicates. Comparisons were made using a two-way ANOVA (**a-d**). Source data

Under fasting conditions or low glycemic diets (such as a ketogenic diet or calorie restriction) that decrease blood glucose levels, ketogenesis occurs predominantly in the liver to produce ketone bodies. Ketone body oxidation then contributes to energy metabolism for extrahepatic tissues, thereby sparing glucose^1^. We and others recently demonstrated that some cancer cells can also metabolize β-hydroxybutyrate (β-OHB)^2–4^. β-OHB oxidation occurs in the mitochondria, where β-OHB dehydrogenase 1 (BDH1), 3-oxoacid CoA-transferase 1 (OXCT1) and a mitochondrial thiolase convert β-OHB into acetyl-CoA, which subsequently enters the tricarboxylic acid (TCA) cycle (Fig. 1a). We assessed BDH1 and OXCT1 protein levels in a panel of cancer cell lines and found three lines that strongly express both enzymes: AL1376, a pancreatic ductal adenocarcinoma (PDAC) cell line derived from the LSL-*Kras*^G12D/+^; *Trp53*^fl/fl^; *Pdx1*-Cre mouse PDAC model^2,5^; B16, a mouse-derived melanoma cell line; and MIA PaCa-2, a human PDAC cell line (Fig. 1b). Although β-OHB is an alternative to glucose for fuelling cellular energy production, we found that β-OHB could not rescue the proliferation defect of glucose-starved cells (Fig. 1c). We then noted that β-OHB is also a source of cytosolic acetyl-CoA. After β-OHB-derived acetyl-CoA is incorporated into citrate in the mitochondria, citrate can be exported into the cytosol and be used to generate cytosolic acetyl-CoA. Cytosolic acetyl-CoA has several downstream fates, including the synthesis of fatty acids, cholesterol, and protein and histone acetylation (Fig. 1a). To test the importance of cytosolic acetyl-CoA production from β-OHB, we took advantage of the observation that fatty acid synthesis is required for cancer cell proliferation when extracellular lipid levels are limiting^6^, and we asked whether β-OHB can rescue the proliferation defect of cells cultured in lipid-depleted media. Indeed, β-OHB promoted cell proliferation in lipid-depleted media across all three cell lines (Fig. 1d). In A549, HeLa and Panc1 cells that had lower BDH1 and/or OXCT1 expression (Fig. 1b), β-OHB did not exhibit this rescue (Extended Data Fig. 1a). Lipid-depleted media lacks both fatty acids and cholesterol, and consistently, the fatty acid synthase (FASN) inhibitor GSK2194069 and the cholesterol synthesis inhibitor simvastatin prevented β-OHB from rescuing the proliferation of lipid-starved cells (Fig. 1e,f). These data suggest that cytosolic acetyl-CoA is an important downstream fate of β-OHB.

To examine the extent to which β-OHB contributes to the cytosolic acetyl-CoA pool, we took advantage of the concept that the labelling pattern of fatty acids such as palmitate by a stable isotope-labelled nutrient precursor directly reflects the fraction of cytosolic acetyl-CoA that is labelled by that precursor^7^. Labelling of palmitate by a stable isotope-labelled nutrient that fully labels the acetyl group in cytosolic acetyl-CoA results in even-numbered isotopomers (M+2, M+4,…M+16) because FASN synthesizes palmitate two carbons at a time using acetyl-CoA-derived malonyl-CoA. Importantly, the abundances of these isotopomers follow a binomial distribution, and at steady-state labelling, this mass isotopomer distribution (MID) reflects the fraction of cytosolic acetyl-CoA that is labelled. The further the MID is shifted to the right towards highly labelled isotopomers, the more cytosolic acetyl-CoA is labelled, and vice versa. Using this principle, a mathematical approach known as isotopomer spectral analysis (ISA) can use the binomial MID of palmitate to calculate the fraction of cytosolic acetyl-CoA that is labelled^7,8^.

We first confirmed that β-OHB is used to synthesize fatty acids by labelling B16, AL1376 and MIA PaCa-2 cells with 5 mM [U-^13^C]-β-OHB for 24 h and assessing fatty acid labelling. β-OHB labelled both saturated fatty acids, such as palmitate (16:0), and monounsaturated fatty acids, such as palmitoleate (16:1(n-7)) and oleate (18:1(n-9)), and this labelling was impaired by the FASN inhibitor GSK2194069 (Extended Data Fig. 1b–d). Moreover, fatty acid labelling was greater in cells grown in lipid-depleted media (Extended Data Fig. 1b–d), reflective of increased fatty acid synthesis. Next, achieving steady-state labelling is important for calculating cytosolic acetyl-CoA labelling by ISA. Given that fatty acids turn over slowly, 2–3 days of labelling are typically needed to approach steady-state labelling^7^. Indeed, we found that the palmitate MID continued shifting to the right between 24 h and 48 h of labelling (Extended Data Fig. 1e–g). Therefore, we conducted all subsequent stable isotope labelling experiments for 48 h.

**Fig. 2:**
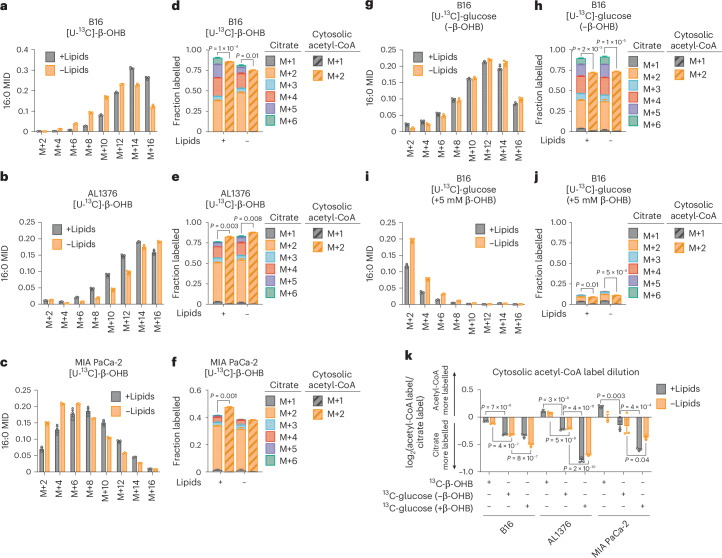
β-OHB is a major source of cytosolic acetyl-CoA. **a**–**c**, Palmitate (16:0) MID for B16 (**a**), AL1376 (**b**) and MIA PaCa-2 (**c**) cells labelled with 5 mM [U-^13^C]-β-OHB for 48 h in lipid-replete versus lipid-depleted culture media. **d**–**f**, Citrate MID (solid bars) and cytosolic acetyl-CoA MID (dashed bars) for B16 (**d**), AL1376 (**e**) and MIA PaCa-2 (**f**) cells labelled with 5 mM [U-^13^C]-β-OHB for 48 h in lipid-replete versus lipid-depleted culture media. **g**–**j**, 16:0 MID (**g**,**i**) and citrate and cytosolic acetyl-CoA MID (**h**,**j**) for B16 cells labelled with 10 mM [U-^13^C]-glucose for 48 h in lipid-replete versus lipid-depleted culture media, either without unlabelled β-OHB (**g**,**h**) or with 5 mM unlabelled β-OHB (**i**,**j**). **k**, Cytosolic acetyl-CoA label dilution from citrate, as calculated by the log_2_(fold change) of the total fraction of cytosolic acetyl-CoA labelled versus the total fraction of citrate labelled, in B16, AL1376 and MIA PaCa-2 cells under the indicated tracing conditions. Data are presented as means; error bars, s.e.m.; *n* = 3 biologically independent replicates. Comparisons were made using a two-tailed Student’s *t*-test (**d**,**e**,**f**,**h**,**j**) or a two-way ANOVA (**k**). Source data

In B16, AL1376 and MIA PaCa-2 cells, we detected substantial labelling of palmitate by 5 mM [U-^13^C]-β-OHB in both lipid-replete and lipid-depleted media (Fig. 2a–c). Notably, at 48 h of labelling, lipid limitation did not robustly increase ^13^C enrichment into palmitate (that is, an upward shift in the MID) (Fig. 2a–c), as was observed when labelling for 24 h (Extended Data Fig. 1b). Given that steady-state labelling reveals the contribution of β-OHB to the palmitate pool, rather than the flux of palmitate synthesis, the lack of an upward shift in the palmitate MID suggests that in both lipid-replete and lipid-depleted conditions, the degree to which β-OHB is used as a fuel for palmitate (versus other carbon sources) is similar. As expected, we also observed ^13^C labelling in citrate, the precursor of cytosolic acetyl-CoA (Fig. 2d–f). Using ISA, we then calculated ^13^C enrichment in cytosolic acetyl-CoA and found that it was highly labelled by β-OHB. Strangely, we noticed that in some cases, such as for AL1376 and MIA PaCa-2 cells, cytosolic acetyl-CoA was labelled to a greater extent than citrate (Fig. 2e,f). This was surprising because labelling of downstream metabolites typically becomes more diluted, rather than enriched, compared to the labelling of their upstream precursors.

**Extended Data Fig. 2:**
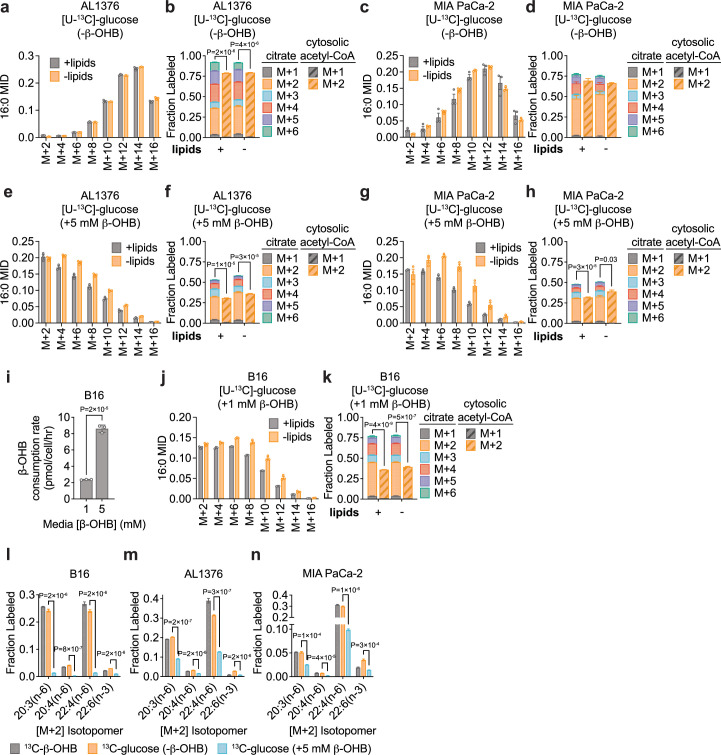
β-OHB is a major source of cytosolic acetyl-CoA. Related to Fig. 2. **a**-**d**, Palmitate (16:0) mass isotopomer distribution (MID) (**a**, **c**) and citrate MID (solid bars) and cytosolic acetyl-CoA MID (dashed bars) (**b, d**) for AL1376 (**a**, **b**) and MIA PaCa-2 (**c**, **d**) cells labeled with 10 mM [U-^13^C]-glucose for 48 h in lipid-replete versus lipid-depleted culture media without unlabeled β-OHB. **e-h**, 16:0 MID (**e**, **g**) and citrate MID (solid bars) and cytosolic acetyl-CoA MID (dashed bars) (**f, h**) for AL1376 (**e**, **f**) and MIA PaCa-2 (**g**, **h**) cells labeled with 10 mM [U-^13^C]-glucose for 48 h in lipid-replete versus lipid-depleted culture media with 5 mM unlabeled β-OHB. **i**, Consumption rates of β-OHB by B16 cells from culture media containing 1 mM or 5 mM β-OHB. **j-k**, 16:0 MID (**j**) and citrate MID (solid bars) and cytosolic acetyl-CoA MID (dashed bars) (**k**) for B16 cells labeled with 10 mM [U-^13^C]-glucose for 48 h in lipid-replete versus lipid-depleted culture media with 1 mM unlabeled β-OHB. **l-n**, [M + 2] fractional labeling of 20:3(n-6), 20:4(n-6), 22:4(n-6), and 22:6(n-3) in B16 (**l**), AL1376 (**m**), and MIA PaCa-2 (**n**) cells labeled with 5 mM [U-^13^C]-β-OHB, 10 mM [U-^13^C]-glucose without unlabeled β-OHB, or 10 mM [U-^13^C]-glucose with 5 mM unlabeled β-OHB for 48 h in lipid-replete media. Data are presented as mean ± s.e.m; *n* = 3 biologically independent replicates. Comparisons were made using a two-tailed Student’s t test (**b**, **d**, **f**, **h**, **i**, **k-n**). Source data

As a comparison, we labelled cells with 10 mM [U-^13^C]-glucose for 48 h and found that in this case, cytosolic acetyl-CoA labelling was always diluted relative to citrate labelling (Fig. 2g,h and Extended Data Fig. 2a–d). This suggests that higher labelling of cytosolic acetyl-CoA relative to citrate is unique to β-OHB and that β-OHB may somehow be more efficiently used to synthesize cytosolic acetyl-CoA compared to glucose. Consistently, adding 5 mM unlabelled β-OHB strongly suppressed 10 mM [U-^13^C]-glucose labelling of palmitate, citrate and cytosolic acetyl-CoA (Fig. 2i,j and Extended Data Fig. 2e–h). Given that 5 mM β-OHB is on the upper end of physiological β-OHB concentrations, it may displace [U-^13^C]-glucose labelling through mass action. In support of this idea, the β-OHB uptake rate by B16 cells was reduced when extracellular β-OHB was lowered from 5 mM to 1 mM, which is more physiological^2,9^ (Extended Data Fig. 2i). The 1 mM β-OHB still substantially displaced [U-^13^C]-glucose labelling of palmitate, citrate and cytosolic acetyl-CoA, but to a lesser extent than 5 mM β-OHB (Extended Data Fig. 2j,k, compare to Fig. 2g–j). Interestingly, we note that 1 mM β-OHB had a larger effect at displacing glucose labelling of cytosolic acetyl-CoA compared to citrate; citrate labelling was reduced by ~15% (from ~90% to ~75%), whereas cytosolic acetyl-CoA labelling was reduced by ~35% (from ~70% to ~35%) (compare Extended Data Fig. 2k to Fig. 2h). These data demonstrate that although the extent to which β-OHB is used for cytosolic acetyl-CoA synthesis depends on its extracellular availability, β-OHB is a major source of cytosolic acetyl-CoA even when glucose is available in excess.

To quantify the degree to which ^13^C labelling was diluted versus enriched in cytosolic acetyl-CoA relative to citrate, we calculated the fold change in labelled cytosolic acetyl-CoA compared to labelled citrate (Fig. 2k). This confirmed that [U-^13^C]-β-OHB highly labelled cytosolic acetyl-CoA, in some cases more than it labelled citrate, whereas [U-^13^C]-glucose labelling of cytosolic acetyl-CoA was always diluted compared to citrate and was further diluted by the presence of unlabelled β-OHB. Finally, we noted that cytosolic acetyl-CoA is also used for fatty acid elongation, including the generation of longer-chain polyunsaturated fatty acids (PUFAs) such as 20:3(n-6), 20:4(n-6), 22:4(n-6) and 22:6(n-3). Indeed, both [U-^13^C]-β-OHB and [U-^13^C]-glucose labelled these PUFAs, and unlabelled β-OHB again suppressed PUFA labelling by [U-^13^C]-glucose (Extended Data Fig. 2l–n).

**Fig. 3:**
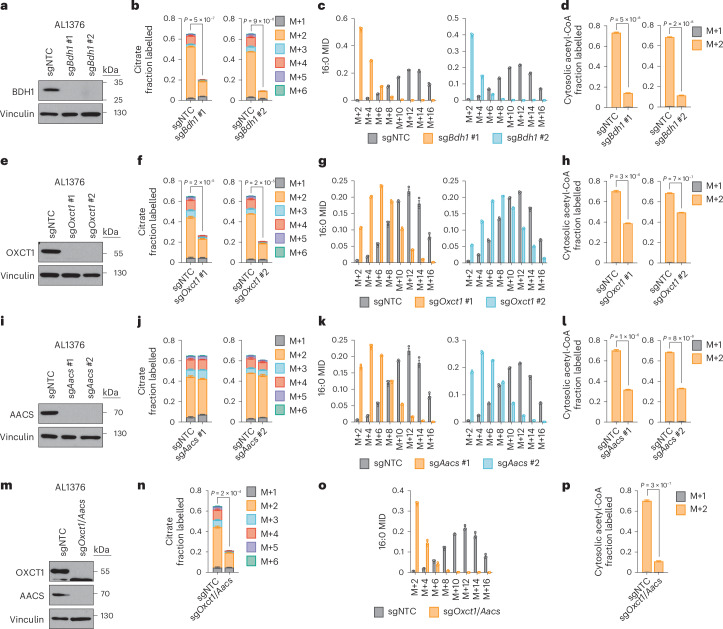
β-OHB can contribute to cytosolic acetyl-CoA production through a citrate-independent route that requires AACS. **a**, Immunoblot for BDH1 and vinculin in the indicated AL1376 knockout lines. NTC, non-targeting control. **b**–**d**, Citrate MID (**b**), palmitate (16:0) MID (**c**) and cytosolic acetyl-CoA MID (**d**) in AL1376 sgNTC, sg*Bdh1* #1 and sg*Bdh1* #2 cells labelled with 5 mM [U-^13^C]-β-OHB for 48 h in lipid-depleted culture media. **e**, Immunoblot for OXCT1 and vinculin in the indicated AL1376 knockout lines. **f**–**h**, Citrate MID (**f**), 16:0 MID (**g**) and cytosolic acetyl-CoA MID (**h**) in AL1376 sgNTC, sg*Oxct1* #1 and sg*Oxct1* #2 cells labelled with 5 mM [U-^13^C]-β-OHB for 48 h in lipid-depleted culture media. **i**, Immunoblot for AACS and vinculin in the indicated AL1376 knockout lines. **j**–**l**, Citrate MID (**j**), 16:0 MID (**k**) and cytosolic acetyl-CoA MID (**l**) in AL1376 sgNTC, sg*Aacs* #1 and sg*Aacs* #2 cells labelled with 5 mM [U-^13^C]-β-OHB for 48 h in lipid-depleted culture media. **m**, Immunoblot for OXCT1, AACS and vinculin in the indicated AL1376 knockout lines. **n**–**p**, Citrate MID (**n**), 16:0 MID (**o**) and cytosolic acetyl-CoA MID (**p**) in AL1376 sgNTC and sg*Oxct1*/*Aacs* cells labelled with 5 mM [U-^13^C]-β-OHB for 48 h in lipid-depleted culture media. Data are presented as means; error bars, s.e.m; *n* = 3 biologically independent replicates. Comparisons were made using a two-tailed Student’s *t-*test (**b**,**d**,**f**,**h**,**j**,**l**,**n**,**p**). Source data

**Extended Data Fig. 3:**
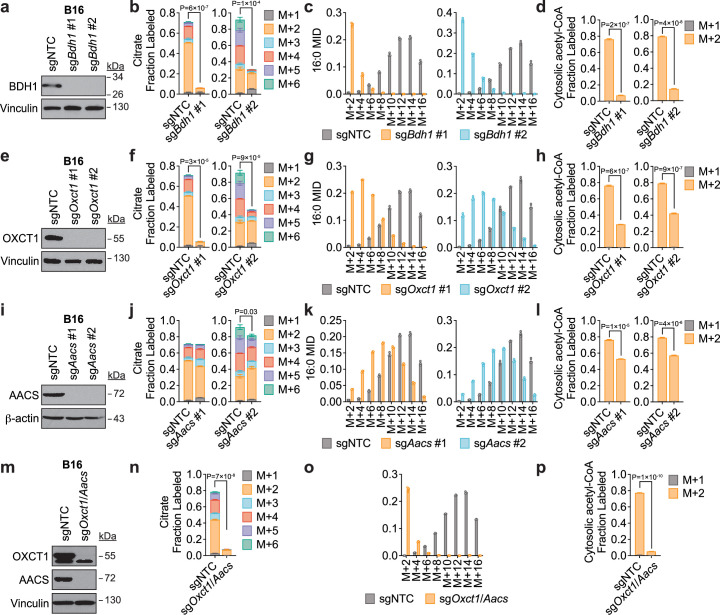
β-OHB can contribute to cytosolic acetyl-CoA production in B16 cells through a citrate-independent route that requires AACS. Related to Fig. 3. **a**, Immunoblot for BDH1 and vinculin in the indicated B16 knockout lines. NTC, non-targeting control. **b-d**, Citrate mass isotopomer distribution (MID) (**b**), palmitate (16:0) MID (**c**), and cytosolic acetyl-CoA MID (**d**) in B16 sgNTC, sg*Bdh1* #1, and sg*Bdh1* #2 cells labeled with 5 mM [U-^13^C]-β-OHB for 48 h in lipid-depleted culture media. **e**, Immunoblot for OXCT1 and vinculin in the indicated B16 knockout lines. **f-h**, Citrate MID (**f**), 16:0 MID (**g**), and cytosolic acetyl-CoA MID (**h**) in B16 sgNTC, sg*Oxct1* #1, and sg*Oxct1* #2 cells labeled with 5 mM [U-^13^C]-β-OHB for 48 h in lipid-depleted culture media. **i**, Immunoblot for AACS and vinculin in the indicated B16 knockout lines. **j-l**, Citrate MID (**j**), 16:0 MID (**k**), and cytosolic acetyl-CoA MID (**l**) in B16 sgNTC, sg*Aacs* #1, and sg*Aacs* #2 cells labeled with 5 mM [U-^13^C]-β-OHB for 48 h in lipid-depleted culture media. **m**, Immunoblot for OXCT1, AACS, and vinculin in the indicated B16 knockout lines. **n-p**, Citrate MID (**n**), 16:0 MID (**o**), and cytosolic acetyl-CoA MID (**p**) in B16 sgNTC and sg*Oxct1*/*Aacs* cells labeled with 5 mM [U-^13^C]-β-OHB for 48 h in lipid-depleted culture media. Data are presented as mean ± s.e.m; *n* = 3 biologically independent replicates. Comparisons were made using a two-tailed Student’s t test (**b**, **d**, **f**, **h**, **j**, **l**, **n**, **p**). Source data

**Extended Data Fig. 4:**
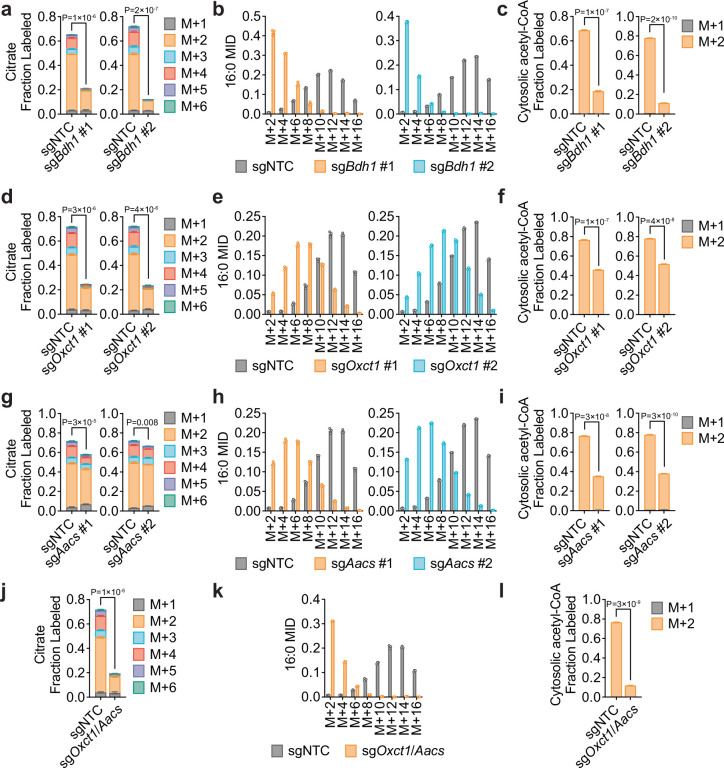
β-OHB can contribute to cytosolic acetyl-CoA production in AL1376 cells through a citrate-independent route that requires AACS in lipid-replete media. Related to Fig. 3. **a**-**c**, Citrate mass isotopomer distribution (MID) (**a**), palmitate (16:0) MID (**b**), and cytosolic acetyl-CoA MID (**c**) in AL1376 sgNTC, sg*Bdh1* #1, and sg*Bdh1* #2 cells labeled with 5 mM [U-^13^C]-β-OHB for 48 h in lipid-replete culture media. **d-f**, Citrate MID (**d**), 16:0 MID (**e**), and cytosolic acetyl-CoA MID (**f**) in AL1376 sgNTC, sg*Oxct1* #1, and sg*Oxct1* #2 cells labeled with 5 mM [U-^13^C]-β-OHB for 48 h in lipid-replete culture media. **g-i**, Citrate MID (**g**), 16:0 MID (**h**), and cytosolic acetyl-CoA MID (**i**) in AL1376 sgNTC, sg*Aacs* #1, and sg*Aacs* #2 cells labeled with 5 mM [U-^13^C]-β-OHB for 48 h in lipid-replete culture media. **j-l**, Citrate MID (**j**), 16:0 MID (**k**), and cytosolic acetyl-CoA MID (**l**) in AL1376 sgNTC and sg*Oxct1*/*Aacs* cells labeled with 5 mM [U-^13^C]-β-OHB for 48 h in lipid-replete culture media. Data are presented as mean ± s.e.m; *n* = 3 biologically independent replicates. Comparisons were made using a two-tailed Student’s t test (**a**, **c**, **d**, **f**, **g**, **i**, **j**, **l**). Source data

**Extended Data Fig. 5:**
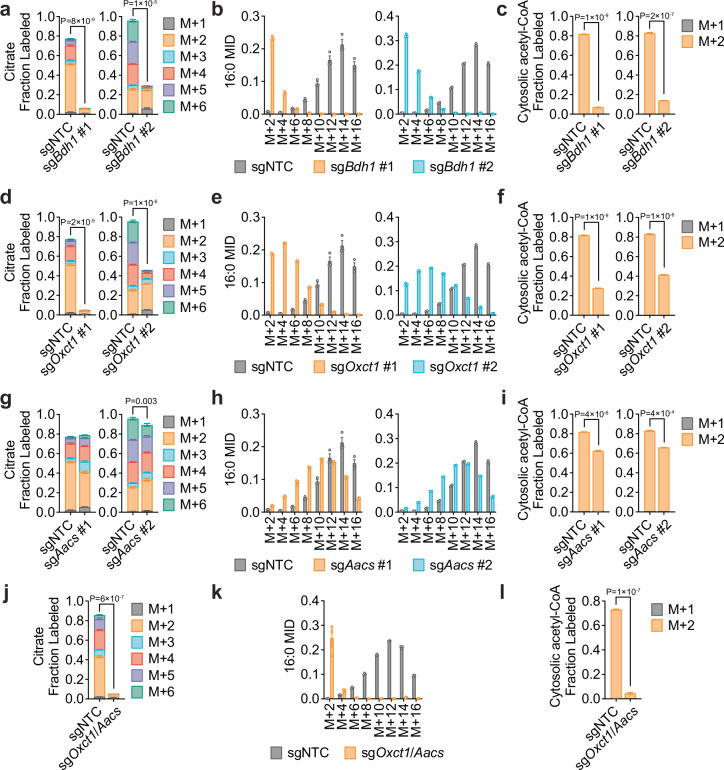
β-OHB can contribute to cytosolic acetyl-CoA production in B16 cells through a citrate-independent route that requires AACS in lipid-replete media. Related to Fig. 3. **a**-**c**, Citrate mass isotopomer distribution (MID) (**a**), palmitate (16:0) MID (**b**), and cytosolic acetyl-CoA MID (**c**) in B16 sgNTC, sg*Bdh1* #1, and sg*Bdh1* #2 cells labeled with 5 mM [U-^13^C]-β-OHB for 48 h in lipid-replete culture media. **d-f**, Citrate MID (**d**), 16:0 MID (**e**), and cytosolic acetyl-CoA MID (**f**) in B16 sgNTC, sg*Oxct1* #1, and sg*Oxct1* #2 cells labeled with 5 mM [U-^13^C]-β-OHB for 48 h in lipid-replete culture media. **g-i**, Citrate MID (**g**), 16:0 MID (**h**), and cytosolic acetyl-CoA MID (**i**) in B16 sgNTC, sg*Aacs* #1, and sg*Aacs* #2 cells labeled with 5 mM [U-^13^C]-β-OHB for 48 h in lipid-replete culture media. **j-l**, Citrate MID (**j**), 16:0 MID (**k**), and cytosolic acetyl-CoA MID (**l**) in B16 sgNTC and sg*Oxct1*/*Aacs* cells labeled with 5 mM [U-^13^C]-β-OHB for 48 h in lipid-replete culture media. Data are presented as mean ± s.e.m; *n* = 3 biologically independent replicates. Comparisons were made using a two-tailed Student’s t test (**a**, **c**, **d**, **f**, **g**, **i**, **j**, **l**). Source data

**Extended Data Fig. 6:**
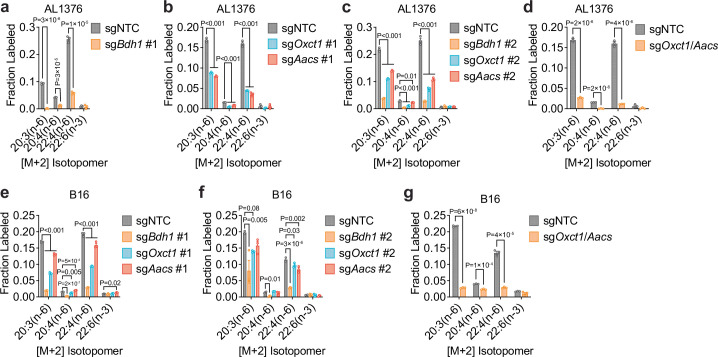
β-OHB contributes to PUFA elongation through both OXCT1 and AACS. Related to Fig. 3. **a**-**d**, [M + 2] fractional labeling of 20:3(n-6), 20:4(n-6), 22:4(n-6), and 22:6(n-3) in the indicated AL1376 knockout cells labeled with 5 mM [U-^13^C]-β-OHB for 48 h in lipid-replete media. **e-g**, [M + 2] fractional labeling of 20:3(n-6), 20:4(n-6), 22:4(n-6), and 22:6(n-3) in the indicated B16 knockout cells labeled with 5 mM [U-^13^C]-β-OHB for 48 h in lipid-replete media. Data are presented as mean ± s.e.m; *n* = 3 biologically independent replicates. Comparisons were made using a two-tailed Student’s t test (**a**, **d**, **g**) or a one-way ANOVA (**b**, **c**, **e**, **f**). Source data

Based on these results, we reasoned that higher labelling of cytosolic acetyl-CoA relative to citrate can only occur if β-OHB is converted to cytosolic acetyl-CoA through a citrate-independent route. To test this idea, we used CRISPR–Cas9 to knock out *Bdh1* or *Oxct1* in AL1376 and B16 cells using two independent single guide RNAs (sgRNAs) (Fig. 3a,e and Extended Data Fig. 3a,e). We then labelled these cells with [U-^13^C]-β-OHB in lipid-depleted media. In *Bdh1* knockout cells, citrate labelling was markedly reduced, as expected (Fig. 3b and Extended Data Fig. 3b). *Bdh1* loss also shifted the palmitate MID almost completely to the left (Fig. 3c and Extended Data Fig. 3c) and thus markedly reduced cytosolic acetyl-CoA labelling (Fig. 3d and Extended Data Fig. 3d). Similar labelling patterns were observed in cells labelled in lipid-replete media (Extended Data Figs. 4a–c and 5a–c). Finally, *Bdh1* loss also suppressed labelling of the omega-6 PUFAs 20:3(n-6), 20:4(n-6) and 22:4(n-6) (Extended Data Fig. 6a,c,e,f). Interestingly, residual labelling of citrate, cytosolic acetyl-CoA, palmitate and PUFAs in *Bdh1* knockout cells suggests that there may be additional enzyme(s) with β-OHB dehydrogenase activity. One possibility is BDH2, although it is uncertain whether BDH2 can convert β-OHB to acetoacetate at physiological β-OHB concentrations because its Michaelis–Menten constant for β-OHB is ~10 mM (ref. ^10^).

In *Oxct1* knockout cells, labelling of citrate from [U-^13^C]-β-OHB was diminished to a similar extent as in *Bdh1* knockout cells, as expected (Fig. 3f and Extended Data Fig. 3f). However, compared to *Bdh1* loss, *Oxct1* loss induced a weaker shift to the left in the palmitate MID (Fig. 3g and Extended Data Fig. 3g; compare to Fig. 3c and Extended Data Fig. 3c) and therefore did not reduce cytosolic acetyl-CoA labelling to the same extent (Fig. 3h and Extended Data Fig. 3h; compare to Fig. 3d and Extended Data Fig. 3d). Cytosolic acetyl-CoA was also labelled to a greater extent than citrate in *Oxct1* knockout cells (compare Fig. 3h with Fig. 3f; Extended Data Fig. 3h with Extended Data Fig. 3f). Similar labelling patterns were observed in cells labelled in lipid-replete media (Extended Data Figs. 4d–f and 5d–f). Finally, *Oxct1* loss suppressed PUFA labelling, but not to the same extent as *Bdh1* loss (Extended Data Fig. 6b,c,e,f). These results confirm that β-OHB can bypass OXCT1-dependent mitochondrial citrate production to generate cytosolic acetyl-CoA.

We reasoned that mitochondrial acetoacetate (the substrate for OXCT1) is probably transported into the cytosol for cytosolic acetyl-CoA production. Acetoacetyl-CoA synthetase (AACS) is a cytosolic enzyme that produces acetoacetyl-CoA from acetoacetate, which can then be converted to cytosolic acetyl-CoA by acetoacetyl-CoA thiolases^11^. AACS is highly expressed in kidney, heart, brain, adipose tissue and osteoclasts^12–14^, and tracer studies with ^14^C-labelled β-OHB have demonstrated cytosolic activation of acetoacetate and its contribution to fatty acid and cholesterol synthesis in rat livers, brain, spinal cord, skin, adipose tissue and lactating mammary glands^15–20^. We therefore asked whether AACS also contributes to cytosolic acetyl-CoA synthesis from β-OHB in cancer cells. We knocked out *Aacs* (Fig. 3i and Extended Data Fig. 3i) and showed that citrate labelling from [U-^13^C]-β-OHB was minimally affected by *Aacs* loss, confirming that mitochondrial β-OHB entry into the TCA cycle remained intact (Fig. 3j and Extended Data Fig. 3j). However, *Aacs* loss shifted the palmitate MID to the left (Fig. 3k and Extended Data Fig. 3k), thus reducing cytosolic acetyl-CoA labelling (Fig. 3l and Extended Data Fig. 3l). Similar labelling patterns were observed in cells labelled in lipid-replete media (Extended Data Figs. 4g–i and 5g–i). Finally, *Aacs* loss also partially impaired PUFA labelling (Extended Data Fig. 6b,c,e,f). These results confirm that β-OHB can contribute to cytosolic acetyl-CoA through a citrate-independent route that requires AACS.

**Extended Data Fig. 7:**
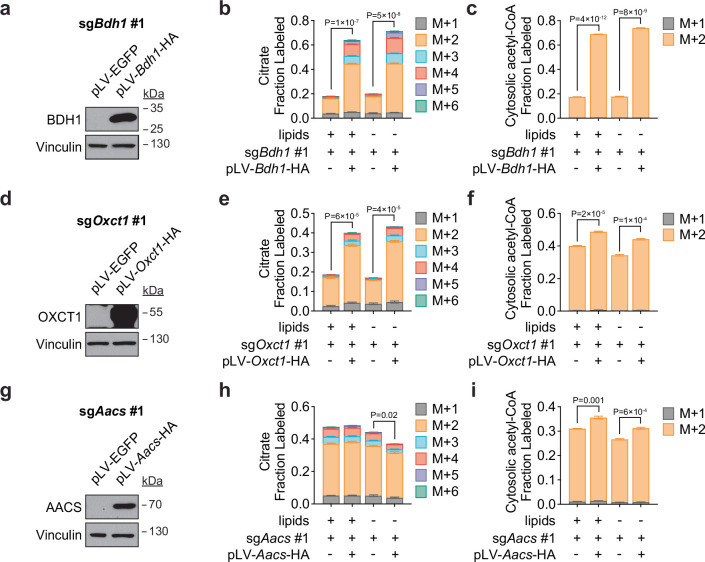
β-OHB can contribute to cytosolic acetyl-CoA production through a citrate-independent route that requires AACS. Related to Fig. 3. **a**, Immunoblot for BDH1 and vinculin in AL1376 sg*Bdh1* #1 cells with or without BDH1-HA re-expression. **b**, **c**, Citrate mass isotopomer distribution (MID) (**b**) and cytosolic acetyl-CoA MID (**c**) in AL1376 sg*Bdh1* #1 cells, with or without BDH1-HA re-expression, labeled with 5 mM [U-^13^C]-β-OHB for 48 h in lipid-replete or lipid-depleted culture media. **d**, Immunoblot for OXCT1 and vinculin in AL1376 sg*Oxct1* #1 cells with or without OXCT1-HA re-expression. **e**, **f**, Citrate MID (**e**) and cytosolic acetyl-CoA MID (**f**) in AL1376 sg*Oxct1* #1 cells, with or without OXCT1-HA re-expression, labeled with 5 mM [U-^13^C]-β-OHB for 48 h in lipid-replete or lipid-depleted culture media. **g**, Immunoblot for AACS and vinculin in AL1376 sg*Aacs* #1 cells with or without AACS-HA re-expression. **h**, **i**, Citrate MID (**h**) and cytosolic acetyl-CoA MID (**i**) in AL1376 sg*Aacs* #1 cells, with or without AACS-HA re-expression, labeled with 5 mM [U-^13^C]-β-OHB for 48 h in lipid-replete or lipid-depleted culture media. Data are presented as mean ± s.e.m; *n* = 3 biologically independent replicates. Comparisons were made using a two-tailed Student’s t test (**b**, **c**, **e**, **f**, **h**, **i**). Source data

We next knocked out both *Oxct1* and *Aacs* (Fig. 3m and Extended Data Fig. 3m), which shifted the palmitate MID almost completely to the left and reduced labelling of citrate, cytosolic acetyl-CoA and PUFAs by [U-^13^C]-β-OHB to the same extent as *Bdh1* loss (Fig. 3n–p and Extended Data Figs. 3n–p, 4j–l, 5j–l and 6d,g). Finally, we also re-expressed *Bdh1*, *Oxct1* and *Aacs* in their respective AL1376 knockout cells (Extended Data Fig. 7a,d,g). Re-expression of *Bdh1* and *Oxct1* restored β-OHB labelling of citrate (Extended Data Fig. 7b,e) and cytosolic acetyl-CoA (Extended Data Fig. 7c,f). *Aacs* re-expression did not increase citrate labelling, but did rescue cytosolic acetyl-CoA labelling (Extended Data Fig. 7h,i). Collectively, these results confirm that both the OXCT1-dependent and the alternative AACS-dependent routes contribute to cytosolic acetyl-CoA production from β-OHB.

**Extended Data Fig. 8:**
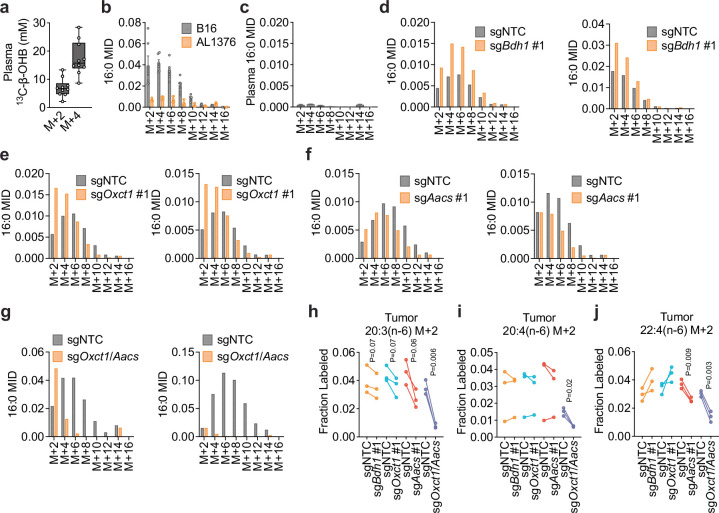
β-OHB contributes to cytosolic acetyl-CoA synthesis through both OXCT1 and AACS in tumors *in vivo*. Related to Fig. 4. **a**, Plasma concentrations of [M + 2] and [M + 4] β-OHB in C57BL/6 J mice infused with [U-^13^C]-β-OHB for 6.5 h. Calculated concentrations were extrapolated from an external standard curve of known β-OHB concentrations. **b**, Palmitate (16:0) mass isotopomer distribution (MID) in subcutaneous B16 or AL1376 tumors in mice infused with [U-^13^C]-β-OHB for 6.5 h. **c**, Plasma 16:0 MID in infused mice. **d-g**, Mice bearing a B16 sgNTC tumor on one flank and a knockout tumor on the other flank were infused with [U-^13^C]-β-OHB for 6.5 h. 16:0 MIDs from each mouse bearing an sgNTC tumor versus a sg*Bdh1* #1 tumor (**d**), sg*Oxct1* #1 tumor (**e**), sg*Aacs* #1 tumor (**f**), and sg*Oxct1*/*Aacs* tumor (**g**). **h-j**, [M + 2] fractional labeling of 20:3(n-6) (**h**), 20:4(n-6) (**i**), and 22:4(n-6) (**j**) in the indicated sgNTC versus knockout tumors. Data are paired between sgNTC and knockout tumors from the same mouse. *n* = 3 biological replicates for each knockout tumor. Data are presented as mean ± s.e.m (**b**, **c**) or as box-and-whisker plots displaying median, interquartile range (boxes), and minima and maxima (whiskers) (**a**). Comparisons were made using a two-tailed paired t test (**h-j**). Source data

Next, we asked whether the AACS-dependent route also occurs in tumours in vivo. Slow lipid turnover makes it challenging to observe fatty acid labelling in in vivo stable isotope labelling experiments. We modified an established infusion protocol^21^ in which mice bearing AL1376 or B16 tumours were fasted for 16 h and then infused with [U-^13^C]-β-OHB through a tail vein catheter with a priming bolus of 478 mg kg^−1^ for 1 min, followed by a constant infusion at 9.75 mg kg^−1^ min^−1^ for 6.5 h. Under these parameters, the concentration of labelled β-OHB in plasma reached supraphysiological levels of >10 mM (Extended Data Fig. 8a), but even with these high concentrations, we only achieved substantial labelling of palmitate to observe its MID in B16, but not AL1376, tumours (Extended Data Fig. 8b). Therefore, we used the B16 model to ask whether the AACS-dependent route operates in vivo.

**Fig. 4:**
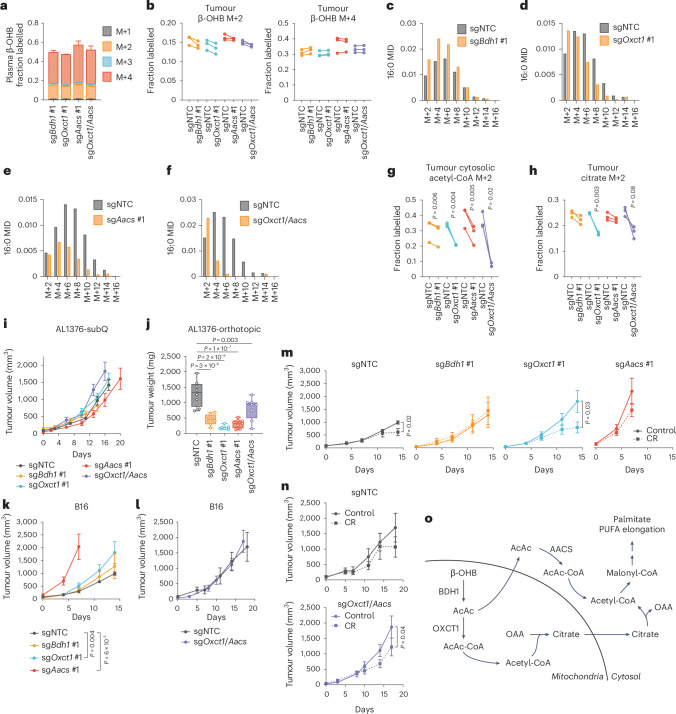
β-OHB metabolism contributes to cytosolic acetyl-CoA synthesis in vivo and supports tumour growth through both OXCT1 and AACS. C57BL/6J mice bearing a B16 sgNTC tumour on one flank and a knockout tumour on the other flank were infused with [U-^13^C]-β-OHB for 6.5 h. **a**, Plasma β-OHB labelling in mice bearing the indicated knockout tumours. *n* = 3 mice for each knockout tumour. **b**, [M+2] and [M+4] fractional labelling of β-OHB in the indicated sgNTC versus knockout tumours. Data are paired between sgNTC and knockout tumours from the same mouse. *n* = 3 biological replicates for each knockout tumour. **c**–**f**, Representative palmitate (16:0) MIDs from *n* = 1 biological replicate of a mouse bearing an sgNTC tumour versus an sg*Bdh1* #1 tumour (**c**), sg*Oxct1* #1 tumour (**d**), sg*Aacs* #1 tumour (**e**) and sg*Oxct1*/*Aacs* tumour (**f**). See Extended Data Fig. 8 for additional biological replicates. **g**,**h**, [M+2] fractional labelling of cytosolic acetyl-CoA (**g**) and citrate (**h**) in the indicated sgNTC versus knockout tumours. Data are paired between sgNTC and knockout tumours from the same mouse. *n* = 3 biological replicates for each knockout tumour. **i**, Tumour volumes of the indicated subcutaneous AL1376 tumours in C57BL/6J male mice. sgNTC *n* = 6 mice, sg*Bdh1* #1 *n* = 4 mice, sg*Oxct1* #1 *n* = 6 mice, sg*Aacs* #1 *n* = 4 mice and sg*Oxct1*/*Aacs* *n* = 5 mice. **j**, Endpoint tumour weights of the indicated AL1376 tumours implanted orthotopically in the pancreas in C57BL/6J male mice. sgNTC *n* = 8 mice, sg*Bdh1* #1 *n* = 7 mice, sg*Oxct1* #1 *n* = 7 mice, sg*Aacs* #1 *n* = 8 mice and sg*Oxct1*/*Aacs* *n* = 8 mice. **k**,**l**, Tumour volumes of the indicated subcutaneous B16 tumours in C57BL/6J male mice. sgNTC *n* = 6 mice, sg*Bdh1* #1 *n* = 6 mice, sg*Oxct1* #1 *n* = 5 mice, sg*Aacs* #1 *n* = 6 mice (**k**) and sgNTC *n* = 3 mice and sg*Oxct1*/*Aacs* *n* = 4 mice (**l**). **m**,**n**, Tumour volumes of the indicated subcutaneous B16 tumours in C57BL/6J male mice exposed to a control or calorie-restricted diet. sgNTC control *n* = 6 mice, CR *n* = 6 mice; sg*Bdh1* #1 control *n* = 6 mice, CR *n* = 6 mice; sg*Oxct1* #1 control *n* = 5 mice, CR *n* = 5 mice; sg*Aacs* #1 control *n* = 6 mice, CR *n* = 6 mice (**m**). sgNTC control *n* = 3 mice, CR n = 4 mice; sg*Oxct1*/*Aacs* control *n* = 4 mice, CR *n* = 4 mice (**n**). **o**, Schematic of β-OHB contribution to cytosolic acetyl-CoA synthesis through both a mitochondrial citrate-dependent route through OXCT1 and a citrate-independent route through AACS. Data are presented as means; error bars, s.e.m (**a**,**i**,**k**–**n**) or as box-and-whisker plots displaying median, interquartile range (boxes) and minima and maxima (whiskers) (**j**). Comparisons were made using a two-tailed paired *t*-test (**g**,**h**), a one-way ANOVA (**j**) or a two-way ANOVA (**i**,**k**–**n**). Source data

C57BL/6J mice were subcutaneously implanted with control B16 single guide non-targeting control (sgNTC) cells on one flank and either B16 sg*Bdh1* #1, sg*Oxct1* #1, sg*Aacs* #1 or sg*Oxct1*/*Aacs* cells on the other flank. After tumours formed, mice were infused with [U-^13^C]-β-OHB, resulting in ~50% ^13^C label enrichment in plasma β-OHB (Fig. 4a). Although [M+4] β-OHB was the predominant isotopomer in the plasma, a smaller fraction was [M+2]-labelled (Fig. 4a). This could potentially result from a ketogenic tissue breaking down [M+4] β-OHB into [M+2] acetyl-CoA, which was then combined with unlabelled acetyl-CoA to generate [M+2] β-OHB that was released into circulation. This highlights a limitation of in vivo tumour tracing studies, in which other tissues metabolize the infused labelled nutrient and transform it into other labelled metabolites that subsequently are available to tumours. Nevertheless, palmitate was minimally labelled in the plasma (Extended Data Fig. 8c), suggesting that any palmitate labelling in tumours probably resulted from fatty acid synthesis by tumour cells.

In tumours, we observed both [M+4] and [M+2] β-OHB labelling for a combined enrichment of ~50% (Fig. 4b). Within each mouse, β-OHB labelling was similar between the sgNTC tumour on one flank versus the knockout tumour on the other flank, enabling direct comparison of labelling patterns in metabolites downstream of β-OHB between the two tumours (Fig. 4b). Surprisingly, *Bdh1* loss did not cause a robust shift to the left in the palmitate MID compared to sgNTC tumours (Fig. 4c and Extended Data Fig. 8d), and [M+2] cytosolic acetyl-CoA and [M+2] citrate labelling were minimally reduced in sg*Bdh1* tumours (Fig. 4g,h). One possible explanation for this may be that [U-^13^C]-β-OHB could also label circulating acetoacetate, which can bypass BDH1 in tumours to synthesize cytosolic acetyl-CoA through OXCT1 or AACS. Alternatively, another enzyme with β-OHB dehydrogenase activity, such as BDH2, may compensate for BDH1 loss. By contrast, we observed a stronger shift to the left in the palmitate MID in sg*Oxct1* and sg*Aacs* tumours, and therefore a stronger decrease in cytosolic acetyl-CoA labelling (Fig. 4d,e,g and Extended Data Fig. 8e,f). As expected, *Oxct1* loss, but not *Aacs* loss, decreased citrate labelling (Fig. 4h). Finally, loss of both *Oxct1* and *Aacs* caused the strongest shift to the left in the palmitate MID (Fig. 4f and Extended Data Fig. 8g), an almost complete loss in cytosolic acetyl-CoA labelling (Fig. 4g) and decreased citrate labelling (Fig. 4h). These results demonstrate that β-OHB-derived cytosolic acetyl-CoA in tumours in vivo is also synthesized through both the mitochondrial citrate-dependent OXCT1 route and the cytosolic citrate-independent AACS route.

Interestingly, we found distinct effects of each gene knockout on PUFA labelling. Labelling of 20:3(n-6) was reduced in all knockout tumours, particularly in sg*Aacs* and sg*Oxct1*/*Aacs* tumours (Extended Data Fig. 8h). By contrast, 20:4(n-6) labelling was reduced only in sg*Oxct1*/*Aacs* tumours (Extended Data Fig. 8i), and 22:4(n-6) labelling was elevated in sg*Bdh1* and sg*Oxct1* tumours but reduced in sg*Aacs* and sg*Oxct1*/*Aacs* tumours (Extended Data Fig. 8j). These results raise the possibility that in tumours in vivo, acetyl-CoA derived from the AACS route may be preferentially used for PUFA elongation, which may be consistent with a recent study suggesting that PUFA elongation by ketones requires AACS^22^.

**Extended Data Fig. 9:**
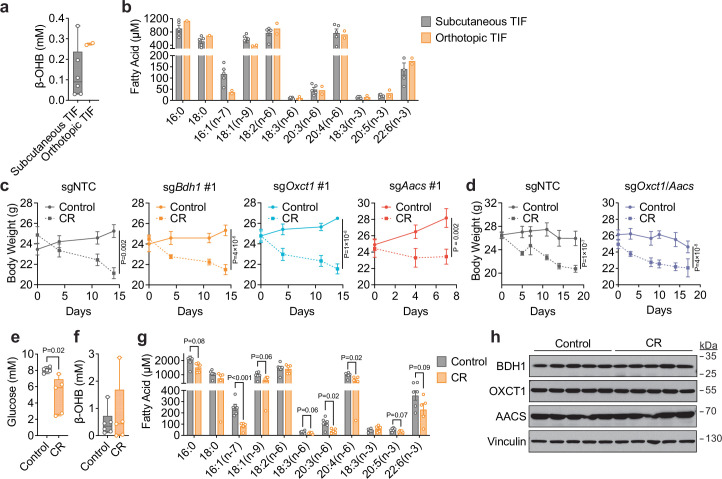
β-OHB is available in the plasma and tumor interstitial fluid in tumor-bearing mice. Related to Fig. 4. **a**, β-OHB concentrations in tumor interstitial fluid (TIF) collected from subcutaneous or orthotopic AL1376 tumors growing in C57BL/6 J mice. Subcutaneous TIF n = 6, Orthotopic TIF n = 2. **b**, Total fatty acid concentrations (free + saponified) in tumor interstitial fluid (TIF) collected from subcutaneous or orthotopic AL1376 tumors growing in mice. Subcutaneous TIF n = 5, Orthotopic TIF n = 2. **c**, **d**, Body weights of mice bearing the indicated subcutaneous B16 tumors exposed to a control or caloric restriction (CR) diet. (**c**) sgNTC Control n = 6 mice, CR n = 6 mice; sg*Bdh1* #1 Control n = 6 mice, CR n = 6 mice; sg*Oxct1* #1 Control n = 5 mice, CR n = 5 mice; sg*Aacs* #1 Control n = 6 mice, CR n = 6 mice. (**d**) sgNTC Control n = 3 mice, CR n = 4 mice; sg*Oxct1*/*Aacs* Control n = 4 mice, CR n = 4 mice. **e-g**, Concentrations of glucose (**e**), β-OHB (**f**), and total fatty acids (free + saponified) (**g**) in the plasma from mice fed a control or CR diet. Control n = 6, CR n = 5. **h**, Immunoblot for BDH1, OXCT1, AACS, and vinculin in B16 sgNTC tumors from mice fed a control or CR diet. Data are presented as mean ± s.e.m (**b-d**, **g**) or as box-and-whisker plots displaying median, interquartile range (boxes), and minima and maxima (whiskers) (**a**, **e**, **f**). Comparisons were made using a two-way ANOVA (**c**, **d**) or a two-tailed Student’s t test (**e**-**g**). Source data

Finally, we asked how β-OHB metabolism through these pathways might influence tumour growth. First, we found that loss of *Bdh1*, *Oxct1*, *Aacs* and *Oxct1*/*Aacs* had no effect on AL1376 PDAC subcutaneous tumour growth (Fig. 4i) but reduced the growth of orthotopic tumours in the pancreas (Fig. 4j). This may suggest that a factor in the pancreatic environment leads to a dependency on ketone metabolism for PDAC tumour growth. β-OHB concentrations averaged ~0.3 mM in tumour interstitial fluid (TIF) isolated from orthotopic tumours, which trended higher than levels found in subcutaneous TIF (Extended Data Fig. 9a), suggesting that β-OHB is available in the tumour microenvironment of orthotopic tumours. Moreover, quantification of total fatty acid levels showed that orthotopic TIF contained lower levels of some fatty acids, specifically monounsaturated fatty acids, compared to subcutaneous TIF (Extended Data Fig. 9b). Although these data do not establish whether impaired fatty acid synthesis from β-OHB was responsible for the slower growth of orthotopic AL1376 tumours lacking *Bdh1*, *Oxct1*, *Aacs* or *Oxct1*/*Aacs*, our results do suggest that ketone metabolism, including through the alternative AACS-dependent route, contributes to PDAC tumour growth in the pancreas.

In subcutaneously implanted B16 tumours, loss of ketone metabolism genes did not impair growth; in fact, loss of either *Oxct1* or *Aacs* alone accelerated tumour growth, which may suggest that ketone metabolism may restrain tumour progression in some contexts (Fig. 4k,l). We asked whether there might be specific physiological contexts under which these ketone metabolism enzymes might be important for facilitating tumour growth. Previously, we demonstrated that caloric restriction (CR) decreases lipid levels and increases β-OHB levels in the tumour microenvironment^2^, and we reasoned that ketone metabolism may be important under these conditions. To test this hypothesis, mice bearing subcutaneous B16 control versus knockout tumours were fed a control diet or CR. As expected, CR reduced mouse body weights (Extended Data Fig. 9c,d) and decreased plasma glucose levels (Extended Data Fig. 9e). β-OHB concentrations in plasma collected from fasted mice under both diets averaged ~0.5 mM, reaching as high as ~3 mM in mice fed a CR diet (Extended Data Fig. 9f). CR also reduced the total plasma concentrations of many fatty acid species (Extended Data Fig. 9g). Finally, CR did not alter BDH1, OXCT1 or AACS protein levels in B16 tumours (Extended Data Fig. 9h).

A dependency on ketone metabolism for tumour growth was revealed under CR conditions. Although the growth of B16 sgNTC and sg*Bdh1* #1 tumours was minimally impaired by CR (Fig. 4m), the growth of B16 tumours lacking OXCT1 was significantly inhibited by CR (Fig. 4m). The growth of B16 sg*Aacs* tumours in CR mice may trend towards a decrease (Fig. 4m) but was difficult to interpret because these tumours reached endpoint size in both diet groups by 7 days after diet administration, in contrast to 14 days for the sgNTC, sg*Bdh1* and sg*Oxct1* tumours. Finally, CR also inhibited the growth of B16 sg*Oxct1*/*Aacs* tumours (Fig. 4n), albeit not to a greater extent than that observed for sg*Oxct1* tumours (Fig. 4m). We note that whether CR inhibited B16 tumour growth appears to correlate with whether loss of the ketone metabolism genes reduced β-OHB-derived cytosolic acetyl-CoA (Fig. 4g), in that *Bdh1* loss did not reduce cytosolic acetyl-CoA labelling and did not sensitize tumours to CR, whereas *Oxct1* or *Oxct1*/*Aacs* loss did lower cytosolic acetyl-CoA labelling and did sensitize tumours to CR. However, the physiological state under which our in vivo tracing experiments were performed was very different from that in CR mice, and our data do not establish whether impaired cytosolic acetyl-CoA synthesis from β-OHB specifically is responsible for improved responses to CR. We also note that the effect sizes observed with B16 knockout tumours were much less than those observed for AL1376 knockout tumours. Nevertheless, these results suggest that ketone metabolism, including through the alternative AACS-dependent route, may have a minor influence on B16 tumour growth in the context of a CR diet. Whether these pathways have a greater contribution to tumour growth in other cancer types or under other contexts will require further study.

In summary, in this study, we demonstrate that in cancer cells capable of metabolizing β-OHB, β-OHB can be a major source for the production of cytosolic acetyl-CoA, even when other key precursors such as glucose are available in excess. In addition to the canonical route of cytosolic acetyl-CoA synthesis through OXCT1-dependent production of citrate from the TCA cycle, we identify an alternative pathway in which β-OHB-derived acetoacetate in mitochondria can be exported to the cytosol and converted into cytosolic acetyl-CoA through AACS and cytosolic thiolases (Fig. 4o). This alternative routing allows β-OHB to bypass oxidation in the mitochondria to support its use as a major contributor to cytosolic acetyl-CoA. This feature distinguishes β-OHB from glucose and glutamine, as carbons from glucose and glutamine need to be routed through citrate to produce cytosolic acetyl-CoA. In this sense, β-OHB is similar to acetate, which is another alternative fuel for tumours that is directly converted into cytosolic acetyl-CoA by ACSS2 (refs. ^23,24^). These results have important implications for understanding how cancer cells maintain their cytosolic acetyl-CoA pool, particularly because ketones are typically absent from most standard culture media.

Notably, two recent studies^22^^,^^25^ have also described the contribution of ketones to fatty acid synthesis through AACS in the liver, highlighting how this alternative pathway functions in multiple tissues and cell types. A distinction between ketone metabolism in liver tissue versus cancer cells is that the liver, as a ketogenic tissue, does not express OXCT1 to prevent ketolysis and ketone oxidation. By contrast, cancer cells that can metabolize ketones can do so through both the OXCT1-dependent and the AACS-dependent routes, and this supports the use of β-OHB as a major contributor to cytosolic acetyl-CoA, even when glucose is present. Of note, our data suggest that the relative contribution of the OXCT1-dependent route versus the AACS-dependent route may be cell-line-dependent. In AL1376 cells, the AACS route appears to be more dominant, given that the shift to the left in the palmitate MID and loss in cytosolic acetyl-CoA labelling was greater in the *Aacs* knockout cells than in the *Oxct1* knockout cells (compare Fig. 3k,l with Fig. 3g,h). Conversely, the OXCT1 route appears to be more dominant in B16 cells (compare Extended Data Fig. 3g,h with Extended Data Fig. 3k,l). The factors that determine which pathway is more dominant and whether the relative activities of these two routes might be regulated in certain contexts remain to be determined.

Finally, cytosolic acetyl-CoA has multiple downstream fates, including fatty acid synthesis^26^, cholesterol synthesis^27^ and protein and histone acetylation^21,28^. How the different routes of cytosolic acetyl-CoA production from β-OHB affect these downstream fates remains an open question, as does whether any of these downstream fates are particularly important for cancer progression under specific contexts. Our identification of an alternative pathway for β-OHB-derived cytosolic acetyl-CoA production will aid future studies that seek to better define how ketone body metabolism influences diverse downstream cellular processes to alter cancer progression.

## Methods

### Cell lines and culture

All validated human cancer cell lines used for this study were obtained from the American Type Culture Association (ATCC), immediately expanded and frozen in multiple aliquots. Thawed cell lines were validated based on known morphology and growth rates. Low passage cells (<30 passages) were used for all experiments. AL1376 PDAC cells were isolated from C57BL/6J *LSL-Kras*^G12D^; *Trp53*^fl/fl^; *Pdx1-*Cre mice^2^. B16 cells were obtained from R. Jones’ laboratory at the Van Andel Institute (VAI). No cell lines used in this study were found in the database of commonly misidentified cell lines that is maintained by the International Cell Line Authentication Committee. Cells were regularly assayed for mycoplasma contamination and passaged for no more than 6 months. All cells were cultured in DMEM (Corning Life Sciences, 10-013-CV) without pyruvate and 10% heat-inactivated dialyzed FBS (Corning Life Sciences, 35-010-CV) unless otherwise specified. For lipid limitation experiments, FBS was stripped of lipids and dialyzed as previously described^29^, and lipid-stripped FBS was added to standard DMEM media, which contains glucose and glutamine but lacks pyruvate (Corning Life Sciences, 10-013-CV), to generate lipid-depleted cell culture media.

### Inhibitors

The FASN inhibitor GSK2194069 (Tocris, 5303) and the cholesterol synthesis inhibitor simvastatin (Cayman Chemical, 10010344) were used at the indicated concentrations.

### Generation of knockout cells by CRISPR–Cas9

sgRNA sequences were chosen from the mouse GeCKO v2 library^30^ and cloned into pSpCas9(BB)-2A-GFP (PX458) (Addgene, 48138). Cells were transfected with these vectors using Lipofectamine 2000 transfection reagent (Invitrogen, 11668027) according to the manufacturer’s protocol. After 48 h, GFP-positive cells were sorted into single cells with a BD FACSymphony S6 cell sorter and grown up as single-cell clones. Knockouts were confirmed by immunoblotting. sgRNA sequences were as follows: *Bdh1 #1*, CGTAGGTCCGACGGGTGTCA; *Bdh1 #2*, AACGCAGGCATCTCAACGTT; *Oxct1* #1, TCTAGGGCACACTTGCCGAG; *Oxct1* #2, ACGAATGATCTCCTCATATG; *Aacs #1*, CGACAGAGTCGCCCTTTACG; and *Aacs #2:* TCCGGTCGTATATGGACTTT.

### Plasmids and generation of stable cDNA-expressing cells

For re-expression of BDH1, OXCT1 and AACS with a carboxy-terminal HA tag, lentivirus vectors pLV[Exp]-Neo-EF1A>hBDH1[NM_203315.3]/HA, pLV[Exp]-Neo-EF1A>hOXCT1[NM_001364300.2]/HA and pLV[Exp]-Neo-EF1A>hAACS[NM_023928.5]/HA were constructed and ordered from VectorBuilder. pLV[Exp]-EGFP/Neo-EF1A>ORF_stuffer was used as an empty vector control. Stable cDNA-expressing cell lines were generated by lentivirus infection for 24 h, followed by selection in DMEM containing 1,000 µg ml^−1^ of G418. After selection, cells were maintained in 400 µg ml^−1^ of G418 until used in experimental assays.

### Immunoblotting

Cells were lysed in radioimmunoprecipitation assay (RIPA) buffer (Thermo Scientific, 89900) supplemented with Halt Protease and Phosphatase Inhibitor Cocktail (ThermoFisher, 78440) for 30 min at 4 °C. Cell extracts were pre-cleared by centrifugation at maximum speed for 15 min at 4 °C, and protein concentration was measured with the Pierce BCA Protein Assay Kit (Thermo Scientific, 23225). Lysates were resolved on SDS–PAGE and transferred electrophoretically to 0.2 µm nitrocellulose membranes (Bio-Rad, 1620112) at 100 V for 60 min. The blots were blocked in Tris-buffered saline buffer (TBST; 10 mmol l^−1^ Tris-HCl pH 8, 150 mmol l^−1^ NaCl and 0.2% Tween-20) containing 5% (w/v) nonfat dry milk for 30 min and then incubated with the specific primary antibody diluted in blocking buffer at 4 °C overnight. Membranes were washed four times in TBST and incubated with horseradish peroxidase (HRP)-conjugated secondary antibody for 1 h at 25 °C. Membranes were washed three times and developed using SuperSignal West Femto Maximum Sensitivity Substrate (Thermo Scientific, 34096). Antibodies were used as follows: BDH1 (Proteintech, 67448-1-Ig, 1:1,000), OXCT1 (Proteintech, 12175-1-AP, 1:1,000), AACS (Proteintech, 13815-1-AP, 1:2,000), Vinculin (Cell Signaling Technology, 137015, clone E1E9V, 1:1,000), β-actin (Cell Signaling Technology, 3700, 1:1,000), anti-mouse IgG HRP-linked secondary antibody (Cell Signaling Technology, 7076, 1:2,000) and anti-rabbit IgG HRP-linked secondary antibody (Cell Signaling Technology, 7074, 1:5,000).

### Proliferation assays

Cells were seeded at an initial density of 20,000–50,000 cells per well on a 24-well plate in 1 ml of DMEM medium. After incubating for 24 h, cells were washed three times with 1 ml of PBS and changed to the indicated growth conditions. To maintain adequate nutrient levels over time, the media was replaced every 24 h over the course of the proliferation assay. Cell confluence was monitored over time using imaging with the Incucyte Live-Cell Analysis System (Sartorius). Doublings per day were calculated by fitting the exponential growth equation to proliferation curves using GraphPad Prism 10.

### β-OHB consumption rate measurements

B16 cells were seeded at an initial density of 25,000 cells per well in a six-well plate in 2 ml of DMEM medium. Additionally, 2 ml of DMEM medium was added to cell-free process blank wells. After incubating for 24 h, the B16 experimental wells and process blank wells were washed three times with 2 ml of PBS. To initiate the assay, 2 ml of DMEM supplemented with 10% FBS, 6 mM glutamine (Life Technologies, 100080) and 1 mM or 5 mM β-OHB (Millipore Sigma, 298360) was added to each well. A total of 1.5 ml of 1 mM and 5 mM β-OHB-containing DMEM was transferred from process blank wells to 1.5 ml Eppendorf tubes and spun down at 100*g* at 4 °C for 5 min. Then, 1 ml of supernatant was transferred to a new 1.5 ml Eppendorf tube and stored at −80 °C; these media samples were used to calculate the initial moles of β-OHB per well. To obtain an initial cell count at the beginning of the consumption assay, mirror wells seeded in parallel with the experimental wells were incubated with 1 ml of 1.62 μM Hoechst (Invitrogen, H1399) solution for 15 min at 25 °C. Wells were then washed two times with 1 ml PBS and incubated in 0.5 ml 4% paraformaldehyde (Life Technologies, 100193) for 15 min at 25 °C. Wells were then washed three times with 1 ml PBS and incubated in 2 ml PBS at 4 °C until imaging.

All experimental and process blank wells were incubated for 60 h to allow for β-OHB depletion and consumption from the media. The volume of media in all experimental and remaining process blank wells was then measured. From these wells, 1.5 ml of media was transferred to a 1.5 ml Eppendorf tube and spun down at 100*g* at 4 °C for 5 min. Then, 1 ml of supernatant was transferred to a new 1.5 ml Eppendorf tube to be stored at −80 °C; these samples were used to calculate the final moles β-OHB per well. Following media collection, experimental wells were incubated in Hoechst dye and fixed with paraformaldehyde as described above.

Media samples were thawed in 25 °C water for 3 min. Then, 20 μl of media from each sample was transferred to a new 1.5 ml Eppendorf tube containing 20 μl of 5 mM [U-^13^C_4_]-β-OHB (Cambridge Isotope Laboratories, CLM-3853). Media and the internal labelled β-OHB standard were co-extracted with extraction buffer consisting of chloroform:methanol (containing 25 mg l^−1^ of butylated hydroxytoluene (Millipore Sigma, B1378)):0.88% KCl (w/v) at a final ratio of 8:4:3. The final extraction buffer also contained 0.75 µg ml^−1^ of norvaline as an additional internal standard used for extraction process normalization. Extracts were vortexed for 15 min and centrifuged at maximum speed (17,000*g*) for 10 min. Polar metabolites (aqueous fraction) were transferred to Eppendorf tubes and dried using a Genevac SpeedVac for further mass spectrometry analysis as described below.

Stained and fixed cells were imaged using a Zeiss Celldiscoverer 7 microscope. Images were stitched using ZEN software (Zeiss), and cell counts were calculated using QuPath (v.0.5.0) with the StarDist plugin for nuclei detection^31^.

β-OHB uptake was calculated using the following equation: consumption rate = −1 × ((pmol β-OHB in media from the final time point) − (pmol β-OHB in media from the initial time point))/(AUC). The area under the growth curve (AUC) was calculated using the following equation: AUC = ((initial cell count)/((doublings per day) × ln2)) × (2^(final time point in days)^ − 1).

### Stable isotope labelling experiments and metabolite extraction

Cells were seeded at an initial density of 70,000–150,000 cells per well in a six-well plate in 2 ml of DMEM medium. After incubating for 24 h, cells were washed three times with 2 ml of PBS and then incubated in the indicated DMEM media without pyruvate for 24 h. For glucose isotope labelling experiments, cells were then cultured with 10 mM [U-^13^C_6_]-glucose (Cambridge Isotope Laboratories, CLM-1396) for 48 h. For β-OHB isotope labelling experiments, cells were cultured with 5 mM [U-^13^C_4_]-β-OHB (Cambridge Isotope Laboratories, CLM-3853) for 48 h.

For each condition, a parallel plate of cells was scanned with an Incucyte Live-Cell Analysis System (Sartorius) and analysed for confluence to normalize extraction buffer volumes based on cell number. An empty well was also extracted as a process control. The extraction buffer consisted of chloroform:methanol (containing 25 mg l^−1^ of butylated hydroxytoluene (Millipore Sigma, B1378)):0.88% KCl (w/v) at a final ratio of 8:4:3. The final extraction buffer also contained 0.75 µg ml^−1^ of norvaline and 0.7 µg ml^−1^ of *cis*-10-heptadecenoic acid as internal standards. For extraction, the medium was aspirated from the cells, and the cells were rapidly washed in ice-cold saline three times. The saline was aspirated, and methanol:0.88% KCl (w/v) (4:3 v/v) was added. Cells were scraped on ice, and the extract was transferred to 1.5 ml Eppendorf tubes (Dot Scientific, RN1700-GMT) before adding chloroform (Supelco, 1.02444). The resulting extracts were vortexed for 15 min and centrifuged at maximum speed (17,000*g*) for 10 min. Polar metabolites (aqueous fraction) were transferred to Eppendorf tubes and dried under nitrogen gas for further analysis. Lipids (organic fraction) were transferred to glass vials (Supelco, 29651-U) and dried under nitrogen gas for further analysis.

### Gas chromatography–mass spectrometry analysis of fatty acids

Fatty acids were analysed as pentafluorobenzyl-fatty acid (PFB-FA) derivatives. Fatty acids were saponified from dried lipid pellets by adding 800 µl of 90% methanol/0.3 M KOH, vortexing and incubating at 80 °C for 60 min. Each sample was then neutralized with 80 µl of formic acid (Supelco, FX0440). Fatty acids were extracted twice with 800 µl of hexane and dried under nitrogen gas. To derivatize, fatty acid pellets were incubated with 100 µl of 10% pentafluorobenzyl bromide (Sigma-Aldrich, 90257) in acetonitrile and 100 µl of 10% *N*,*N*-diisopropylethylamine (Sigma-Aldrich, D125806) in acetonitrile at 25 °C for 30 min. PFB-FA derivatives were dried under nitrogen gas and resuspended in 50 µl of hexane for gas chromatography–mass spectrometry (GC–MS) analysis.

GC–MS was conducted with a TRACE TR-FAME column (ThermoFisher, 260M154P) installed in a Thermo Scientific TRACE 1600 gas chromatograph coupled to a Thermo ISQ 7610 mass spectrometer. Helium was used as the carrier gas at a constant flow of 1.8 ml min^−1^. A 1 µl sample was injected at 250 °C at a 4:1 split (for total lipid extracts) or splitless mode (for 3PLE extracts). After injection, the GC oven was held at 100 °C for 0.5 min, increased to 200 °C at 40 °C min^−1^, held at 200 °C for 1 min, increased to 250 °C at 5 °C min^−1^ and held at 250 °C for 11 min. The MS system operated under negative chemical ionization mode with methane gas at a flow rate of 1.25 ml min^−1^, and the MS transfer line and ion source were held at 255 °C and 200 °C, respectively. The detector was used in scanning mode with an ion range of 150–500 *m/z*. Total ion counts were determined by integrating appropriate ion fragments for each PFB-FA^32^ using Skyline software^33^. Metabolite data were normalized to the internal standard and background-corrected using a process blank sample. Mass isotopologue distributions were corrected for natural abundance using IsoCorrectoR^34^. Cytosolic acetyl-CoA labelling was calculated from the palmitate MID using ISA from FAMetA^8^. Absolute concentrations of fatty acids were calculated based on an external standard curve using the Supelco 37 Component FAME Mix (Millipore Sigma, CRM47885).

### GC–MS analysis of polar metabolites

Polar metabolites were analysed as MOX-TBDMS derivatives. Dried and frozen metabolite extracts were derivatized with 16 µl of MOX reagent (Thermo Fisher TS-45950) for 60 min at 37 °C, followed by derivatization with 20 µl of *N*-tert-butyldimethylsilyl-*N*-methyltrifluoroacetamide with 1% tert-butyldimethylchlorosilane (Millipore Sigma, 375934) for 30 min at 60 °C. Derivatized samples were analysed by GC–MS, using a DB-5MS column (Agilent Technologies, 122-3832) installed in an Agilent 7890B gas chromatograph coupled to an Agilent 5977B mass spectrometer. Helium was used as the carrier gas at a constant flow rate of 1.2 ml min^−1^. A 1 µl sample was injected in split mode (1:4) at 320 °C. After injection, the GC oven was held at 95 °C for 1 min, increased to 118 °C at 40 °C min^−1^, held at 118 °C for 2 min, increased to 250 °C at 12 °C min^−1^, ramped to 320 °C at 40 °C min^−1^ and held at 320 °C for 7 min. The MS system operated under electron impact ionization at 70 eV, and the MS source and quadrupole were held at 230 °C and 150 °C, respectively. The detector was used in scanning mode with an ion range of 50–800 *m*/*z*. Total ion counts were determined by integrating appropriate ion fragments for each metabolite using Skyline software^33^. Metabolite data were normalized to the internal standard, and mass isotopologue distributions were corrected for natural abundance using IsoCorrectoR^34^. Absolute quantification of β-OHB was calculated based on either an external standard curve or on the known concentrations of isotopically labelled internal standards.

Glucose was analysed by GC–MS as described previously^35^. Dried and frozen metabolite extracts were derivatized with 65 µl of 2% (w/v) hydroxylamine hydrochloride (Millipore Sigma, 255580) in pyridine (Millipore Sigma, PX2012-7) for 60 min at 90 °C, followed by derivatization with 130 µl of propionic anhydride (Millipore Sigma, 240311) for 30 min at 60 °C. Derivatized samples were then dried in a Genevac SpeedVac and resuspended in 100 µl of ethyl acetate (Millipore Sigma, 1.03649) and transferred to glass GC–MS vials. Samples were analysed by GC–MS as described above, except helium was used as the carrier gas at a constant flow rate of 0.88 ml min^−1^, and 1 µl of sample was injected at a 10:1 split mode at 250 °C. After injection, the GC oven was held at 80 °C for 1 min, ramped to 280 °C at 20 °C min^−1^ and held at 280 °C for 4 min. Absolute quantification of glucose was calculated based on an external standard curve.

### Animal studies

All experiments conducted in this study were approved by the VAI Institutional Animal Care and Use Committee (IACUC). For subcutaneous tumour growth, a maximum tumour burden of 2 cm^3^ was permitted per IACUC protocol, and these limits were not exceeded. Male C57BL/6J mice (3–4 months old) were used in this study. All animals were housed at ambient temperature and humidity (18–23 °C, 40–60% humidity) with a 12 h light and 12 h dark cycle and co-housed with littermates with ad libitum access to water, unless otherwise stated. All experimental groups were age-matched, numbered and randomly assigned based on treatment, and experiments were conducted in a blinded manner. Data were collected from distinct animals, where *n* represents biologically independent samples. Statistical methods were not used to pre-determine sample size.

For subcutaneous tumours, C57BL/6J mice (The Jackson Laboratory 000664 or internal mouse colony) were subcutaneously injected with 2 × 10^5^ mouse AL1376 cells or 7 × 10^5^ mouse B16 cells into both flanks in 100 µl of PBS per injection. For orthotopic PDAC tumours, mice were injected with 2 × 10^5^ AL1376 cells into the pancreas in 20 µl of a 1:1 mixture of PBS and Matrigel (Corning, 356234) per injection, as previously described^36,37^. All mice were administered a modified AIN-93G control diet (Envigo TD.97184) as the control diet during tumour formation, and 7–9 days after cell injection, when palpable tumours had formed, animals were randomly placed into different diet groups. Mice were weighed before the start of diet administration to ensure that different cohorts had similar starting body weights, and body weights were also measured over the course of each experiment. Subcutaneous tumour volume was determined using (π/6)(*W*^2^)(*L*), where *W* represents width and *L* represents length as measured by callipers. Orthotopic tumours were dissected at the endpoint and weighed. At the end of each experiment, animals were killed, blood was collected by orbital bleed, TIF was collected as previously described^38^ and tumours were rapidly collected, weighed and freeze-clamped in liquid nitrogen.

### Tumour, plasma and TIF metabolite extraction

For tumours, frozen tissues were ground into powder using a mortar and pestle. Tissue powder was then weighed into glass vials (Supelco, 29651-U). Blood collected from animals was immediately placed in EDTA tubes (Sarstedt 41.1395.105) and centrifuged to separate plasma. TIF was collected as previously described^38^. Metabolites were extracted with extraction buffer consisting of chloroform:methanol (containing 25 mg l^−1^ of butylated hydroxytoluene (Millipore Sigma, B1378)):0.88% KCl (w/v) at a final ratio of 8:4:3. The final extraction buffer also contained 0.75 µg ml^−1^ of norvaline and 0.7 µg ml^−1^ of *cis*-10-heptadecenoic acid as internal standards. Extracts were vortexed for 15 min and centrifuged at maximum speed (17,000*g*) for 10 min. Polar metabolites (aqueous fraction) were transferred to Eppendorf tubes and dried in a Genevac SpeedVac for further MS analysis as described above. Lipids (organic fraction) were transferred to glass vials (Supelco, 29651-U) and dried under nitrogen gas for further MS analysis as described above.

### In vivo [U-^13^C]-β-OHB infusions

In vivo infusions of [U-^13^C]-β-OHB were conducted using previously described protocols^21^. In brief, tumour-bearing mice were fasted for 16 h before being infused with [U-^13^C]-β-OHB (Cambridge Isotope Laboratories, CLM-3853) through a tail vein catheter. Mice received an initial bolus of 478 mg kg^−1^ for 1 min, followed by infusion of 0.05 μl min^−1^ g^−1^ using a 1.5 M stock concentration for 6.5 h. Subsequently, mice were cervically dislocated, and tumours were freeze-clamped in liquid nitrogen. Blood was collected by orbital bleed, mixed with EDTA, centrifuged at 3,000 RCF for 10 min at 4 °C and plasma was snap-frozen in liquid nitrogen. Frozen tumours were crushed into a fine powder using a chilled mortar and pestle and stored at −80 °C. Between 5 mg and 20 mg of powdered tumour was measured for metabolite extraction.

### Animal diets

A modified AIN-93G diet (Envigo TD.97184) was used as the control diet. The 40% CR diet (Envigo TD.210722) was formulated by modifying the control diet, as previously described^2^. For CR studies, mice were individually housed. Before diet administration, the average daily consumption (by weight) of the control diet was determined. Upon experimental diet initiation, control mice were fed daily with the determined average daily food consumption weight, and CR mice were fed daily with a food weight corresponding to 40% of the control caloric consumption.

### Statistics and reproducibility

Sample sizes, reproducibility and statistical tests used for each figure are denoted in the figure legends. No statistical methods were used to pre-determine sample sizes, but our sample sizes are similar to those reported in previous publications^2,39^. Data distribution was assumed to be normal, but this was not formally tested. All graphs were generated using GraphPad Prism 10.

### Reporting summary

Further information on research design is available in the Nature Portfolio Reporting Summary linked to this article.

## Supplementary information

Reporting Summary

## Source data

Source Data Fig. 1 Statistical source data for Fig. 1.

Source Data Fig. 2 Statistical source data for Fig. 2.

Source Data Fig. 3 Statistical source data for Fig. 3.

Source Data Fig. 4 Statistical source data for Fig. 4.

Source Data Fig. 1 Blots Unprocessed western blots for Fig. 1.

Source Data Fig. 3 Blots Unprocessed western blots for Fig. 3.

Source Data Extended Data Fig. 1 Statistical source data for Extended Data Fig. 1.

Source Data Extended Data Fig. 2 Statistical source data for Extended Data Fig. 2.

Source Data Extended Data Fig. 3 Statistical source data for Extended Data Fig. 3.

Source Data Extended Data Fig. 4 Statistical source data for Extended Data Fig. 4.

Source Data Extended Data Fig. 5 Statistical source data for Extended Data Fig. 5.

Source Data Extended Data Fig. 6 Statistical source data for Extended Data Fig. 6.

Source Data Extended Data Fig. 7 Statistical source data for Extended Data Fig. 7.

Source Data Extended Data Fig. 8 Statistical source data for Extended Data Fig. 8.

Source Data Extended Data Fig. 9 Statistical source data for Extended Data Fig. 9.

Source Data Extended Data Fig. 3 Blots Unprocessed western blots for Extended Data Fig. 3.

Source Data Extended Data Fig. 7 Blots Unprocessed western blots for Extended Data Fig. 7.

Source Data Extended Data Fig. 9 Blots Unprocessed western blots for Extended Data Fig. 9.

## Data Availability

All data generated and analysed during this study are included in this published article and in Source Data for Figs. 1–4 and Extended Data Figs. 1–9. Correspondence and requests for materials should be addressed to the corresponding author. Source data are provided with this paper.
