# Supplementary material for: An alternative route for β-hydroxybutyrate metabolism supports cytosolic acetyl-CoA synthesis in cancer cells
Source: Nat Metab. 2025 Sep 8;7(10):2033–44. doi: 10.1038/s42255-025-01366-y (PMC12552118; doi:10.1038/s42255-025-01366-y)
Supplement: Supplementary file 1 — Reporting Summary [file 42255_2025_1366_MOESM1_ESM.pdf]

nature portfolio

Corresponding author(s): Evan Lien

Last updated by author(s): May 20, 2025

Reporting Summary

Nature Portfolio wishes to improve the reproducibility of the work that we publish. This form provides structure for consistency and transparency in reporting. For further information on Nature Portfolio policies, see our Editorial Policies and the Editorial Policy Checklist.

Statistics

For all statistical analyses, confirm that the following items are present in the figure legend, table legend, main text, or Methods section.

n/a	Confirmed	
☐	☒	The exact sample size (*n*) for each experimental group/condition, given as a discrete number and unit of measurement
☐	☒	A statement on whether measurements were taken from distinct samples or whether the same sample was measured repeatedly
☐	☒	The statistical test(s) used AND whether they are one- or two-sided<br>*Only common tests should be described solely by name; describe more complex techniques in the Methods section.*
☒	☐	A description of all covariates tested
☒	☐	A description of any assumptions or corrections, such as tests of normality and adjustment for multiple comparisons
☐	☒	A full description of the statistical parameters including central tendency (e.g. means) or other basic estimates (e.g. regression coefficient) AND variation (e.g. standard deviation) or associated estimates of uncertainty (e.g. confidence intervals)
☐	☒	For null hypothesis testing, the test statistic (e.g. *F*, *t*, *r*) with confidence intervals, effect sizes, degrees of freedom and *P* value noted<br>*Give P values as exact values whenever suitable.*
☒	☐	For Bayesian analysis, information on the choice of priors and Markov chain Monte Carlo settings
☒	☐	For hierarchical and complex designs, identification of the appropriate level for tests and full reporting of outcomes
☒	☐	Estimates of effect sizes (e.g. Cohen's *d*, Pearson's *r*), indicating how they were calculated

Our web collection on statistics for biologists contains articles on many of the points above.

Software and code

Policy information about availability of computer code

Data collection	Gas chromatography-mass spectrometry data were collected using commercial Agilent MassHunter and Thermo Scientific Chromeleon.
Data analysis	Mass spectrometry data were analyzed using open-source Skyline v24.1 software from MacCoss Lab Software. Mass isotopologue distributions were corrected for natural abundance using IsoCorrectoR, which has been previously published. Cytosolic acetyl-CoA labeling was calculated using isotopomer spectral analysis through FAMetA scripts, which have been previously published. All graphs were generated using commercial Graphpad Prism version 10.3.1.

For manuscripts utilizing custom algorithms or software that are central to the research but not yet described in published literature, software must be made available to editors and reviewers. We strongly encourage code deposition in a community repository (e.g. GitHub). See the Nature Portfolio guidelines for submitting code & software for further information.

Data

Policy information about availability of data

All manuscripts must include a data availability statement. This statement should provide the following information, where applicable:

- Accession codes, unique identifiers, or web links for publicly available datasets
- A description of any restrictions on data availability
- For clinical datasets or third party data, please ensure that the statement adheres to our policy

All data generated and analyzed during this study are included in this published article. Correspondence and requests for materials should be addressed to Evan C. Lien (evan.lien@vai.org).

Research involving human participants, their data, or biological material

Policy information about studies with human participants or human data. See also policy information about sex, gender (identity/presentation), and sexual orientation and race, ethnicity and racism.

Reporting on sex and gender	N/A
Reporting on race, ethnicity, or other socially relevant groupings	N/A
Population characteristics	N/A
Recruitment	N/A
Ethics oversight	N/A

Note that full information on the approval of the study protocol must also be provided in the manuscript.

Field-specific reporting

Please select the one below that is the best fit for your research. If you are not sure, read the appropriate sections before making your selection.

☒ Life sciences ☐ Behavioural & social sciences ☐ Ecological, evolutionary & environmental sciences

For a reference copy of the document with all sections, see nature.com/documents/nr-reporting-summary-flat.pdf

Life sciences study design

All studies must disclose on these points even when the disclosure is negative.

Sample size	Statistical methods were not performed to pre-determine sample size for animal studies. The number of animals assigned per condition was selected empirically to account for the variability of the examined phenotypes based on prior experience with the model, as described in refs. 4, 23. For tissue culture experiments, statistical methods were not performed to pre-determine sample sizes. All tissue culture experiments were performed with a minimum of three independent biological replicates, which were sufficient to detect significant differences.
Data exclusions	No data were excluded from the analyses.
Replication	All attempts at replication were successful. All tissue culture experiments were replicated with a minimum of three independent biological replicates (3 replicates on different days).
Randomization	All experimental groups for animal studies were age-matched, numbered, and randomly assigned to different treatments. For all animal studies, mice were injected with mouse-derived melanoma or PDAC cells to develop subcutaneous or orthotopic tumors. After tumors were palpable, tumor volume and mouse body weight were measured to ensure that all groups of mice had similar starting tumor volume and body weight. For tissue culture experiments, random allocation was not applicable because each cell line was considered independently.
Blinding	Measurements for all animal experiments were conducted in a blinded manner. For tissue culture experiments, investigators were not blinded to group allocation during data collection and analysis because experimental set up involved visible identification labels of tissue culture wells indicating drug treatments and media conditions.

Reporting for specific materials, systems and methods

We require information from authors about some types of materials, experimental systems and methods used in many studies. Here, indicate whether each material, system or method listed is relevant to your study. If you are not sure if a list item applies to your research, read the appropriate section before selecting a response.

Materials & experimental systems

n/a	Involved in the study
☐	☒ Antibodies
☐	☒ Eukaryotic cell lines
☒	☐ Palaeontology and archaeology
☐	☒ Animals and other organisms
☒	☐ Clinical data
☒	☐ Dual use research of concern
☒	☐ Plants

Methods

n/a	Involved in the study
☒	☐ ChIP-seq
☒	☐ Flow cytometry
☒	☐ MRI-based neuroimaging

Antibodies

Antibodies used	Antibodies were used as follows: BDH1 (Proteintech, 67448-1-Ig, 1:1000), OXCT1 (Proteintech 12175-1-AP, 1:1000), AACS (Proteintech, 13815-1-AP, 1:2000), Vinculin (Cell Signaling Technology 137015, clone E1E9V, 1:1000), β-actin (Cell Signaling Technology 3700, 1:1000), anti-mouse IgG HRP-linked secondary antibody (Cell Signaling Technology 7076, 1:2000), and anti-rabbit IgG HRP-linked secondary antibody (Cell Signaling Technology 7074, 1:5000).
Validation	Validation of primary antibodies:<br><br>BDH1 (Proteintech, 67448-1-Ig): specificity of this antibody is validated by (1) immunoblot analysis of BDH1 knockout cells generated in this study, and (2) data on the vendor's website: https://www.ptglab.com/products/BDH1-Antibody-67448-1-Ig.htm<br><br>OXCT1 (Proteintech 12175-1-AP): specificity of this antibody is validated by (1) immunoblot analysis of OXCT1 knockout cells generated in this study, and (2) data on the vendor's website: https://www.ptglab.com/products/SCOT-Antibody-12175-1-AP.htm<br><br>AACS (Proteintech, 13815-1-AP): specificity of this antibody is validated by (1) immunoblot analysis of AACS knockout cells generated in this study, and (2) data on the vendor's website: https://www.ptglab.com/products/AACS-Antibody-13815-1-AP.htm<br><br>Vinculin (Cell Signaling Technology 13901, clone E1E9V): specificity of this antibody is well-validated by data on the vendor's website: https://www.cellsignal.com/products/primary-antibodies/vinculin-e1e9v-xp-rabbit-mab/13901<br><br>beta-actin (Cell Signaling Technology 3700): specificity of this antibody is well-validated by data on the vendor's website: https://www.cellsignal.com/products/primary-antibodies/b-actin-8h10d10-mouse-mab/3700

Eukaryotic cell lines

Policy information about cell lines and Sex and Gender in Research

Cell line source(s)	AL1376 pancreatic ductal adenocarcinoma cells were isolated from C57BL/6J LSL-Kras(G12D);Trp53fl/fl;Pdx1-Cre mice as previously described. B16 cells were obtained from the laboratory of Russell G. Jones. MIA PaCa-2, HeLa, Panc1, and A549 cells were obtained from the American Type Culture Collection (ATCC).
Authentication	Cell lines used were not authenticated.
Mycoplasma contamination	All cell lines were routinely tested for mycoplasma contamination and were confirmed to be negative.
Commonly misidentified lines<br>(See ICLAC register)	No commonly misidentified cell lines were used in these studies.

Animals and other research organisms

Policy information about studies involving animals; ARRIVE guidelines recommended for reporting animal research, and Sex and Gender in Research

Laboratory animals	For all animal (Mus musculus) studies, 10-16 week old male C57BL/6J mice (The Jackson Laboratory 000664) were used. Mice were housed at ambient temperature and humidity (18-23°C, 40-60% humidity).
Wild animals	The study did not involve wild animals.
Reporting on sex	All animal studies were performed in male mice. This study does not involve sex-dependent differences.
Field-collected samples	This study did not involve field-collected samples.
Ethics oversight	All experiments conducted in this study were approved by the VAI IACUC.

Note that full information on the approval of the study protocol must also be provided in the manuscript.

Plants

Seed stocks	N/A
Novel plant genotypes	N/A
Authentication	N/A
