# Supplementary figures and images for: An alternative route for β-hydroxybutyrate metabolism supports cytosolic acetyl-CoA synthesis in cancer cells
Source: Nat Metab. 2025 Sep 8;7(10):2033–44. doi: 10.1038/s42255-025-01366-y (PMC12552118; doi:10.1038/s42255-025-01366-y)

Source Data Figure 1b. Uncropped western blot images.

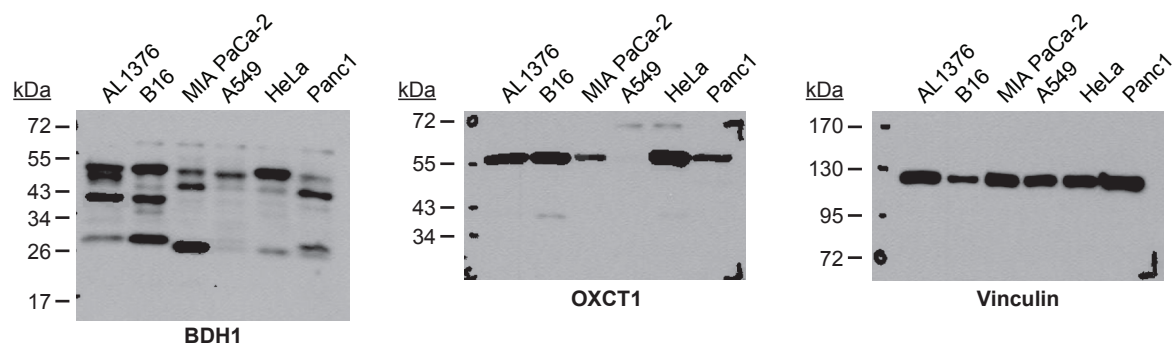

Supplement: Supplementary file 6 — Unprocessed western blots for Fig. 1. [file 42255_2025_1366_MOESM6_ESM.pdf]

Source Data Figure 3. Uncropped western blot images.

Figure 3a

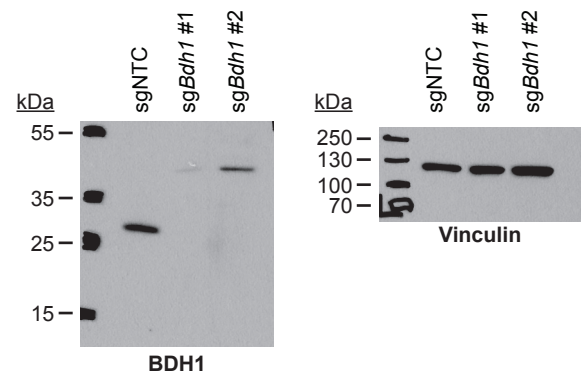

Figure 3e

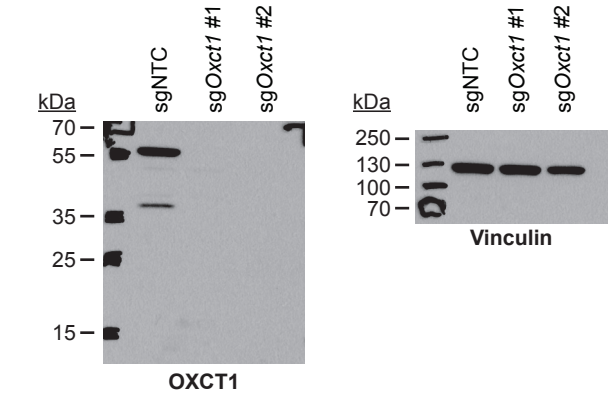

Figure 3i

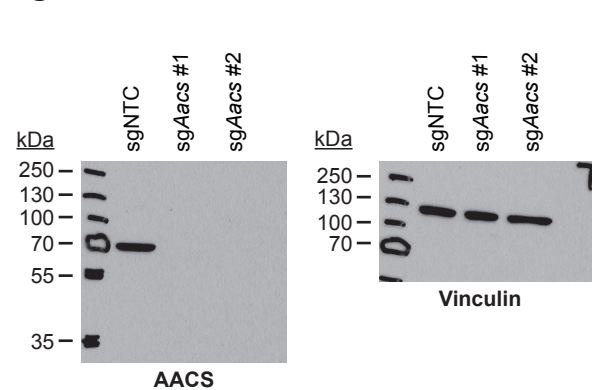

Figure 3m

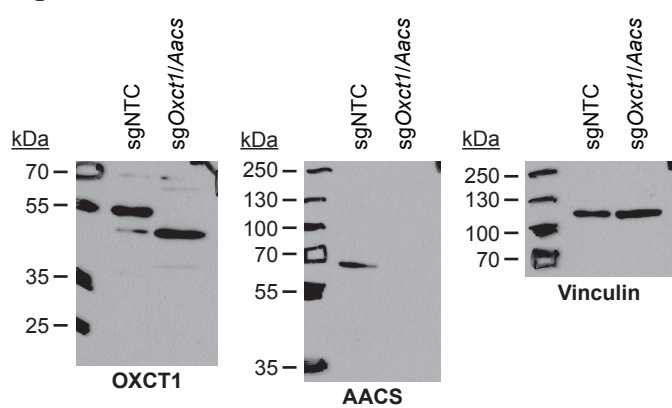

Supplement: Supplementary file 7 — Unprocessed western blots for Fig. 3. [file 42255_2025_1366_MOESM7_ESM.pdf]

Source Data Extended Data Figure 9h. Uncropped western blot images.

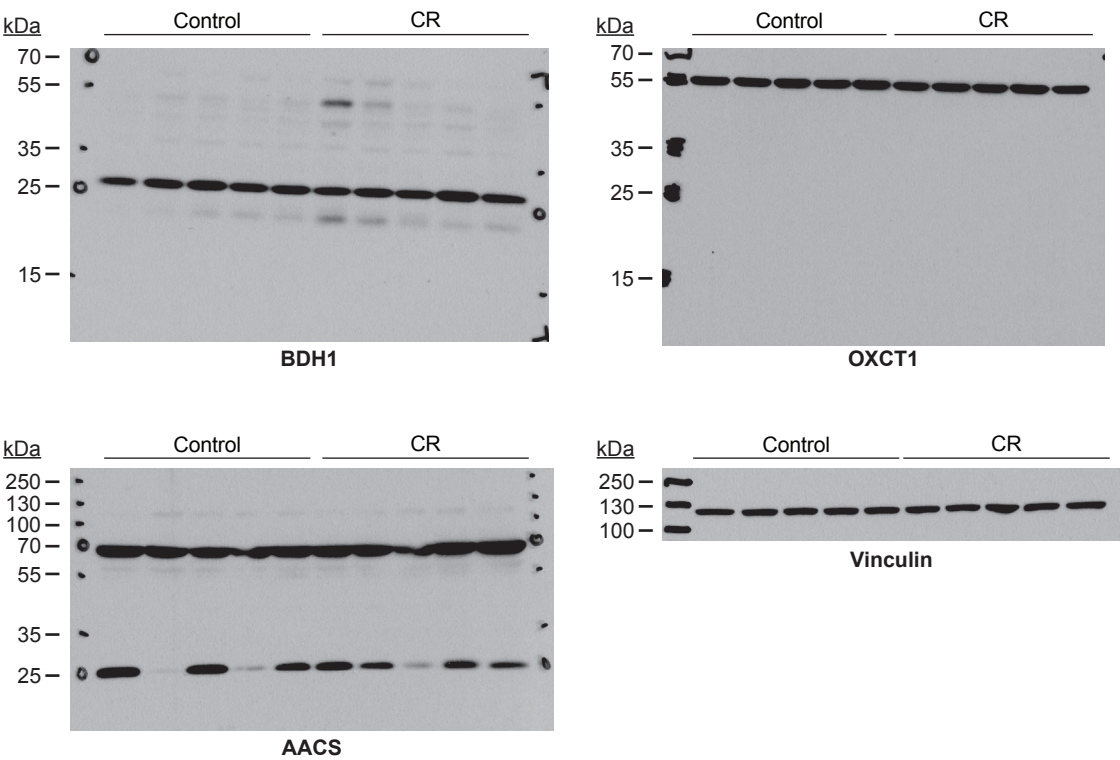

Supplement: Supplementary file 19 — Unprocessed western blots for Extended Data Fig. 9. [file 42255_2025_1366_MOESM19_ESM.pdf]
