# Supplementary material for: An alternative route for β-hydroxybutyrate metabolism supports cytosolic acetyl-CoA synthesis in cancer cells
Source: Nat Metab. 2025 Sep 8;7(10):2033–44. doi: 10.1038/s42255-025-01366-y (PMC12552118; doi:10.1038/s42255-025-01366-y)

Source Data Extended Data Figure 3. Uncropped western blot images.

ED Figure 3a

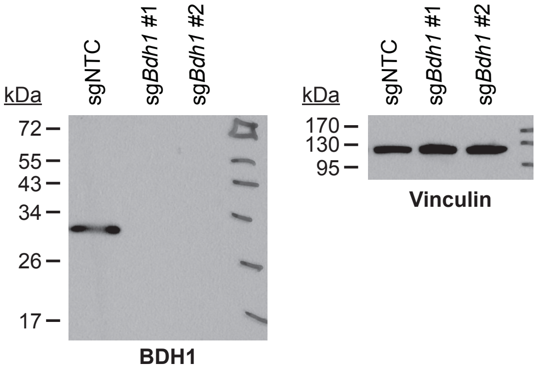

ED Figure 3e

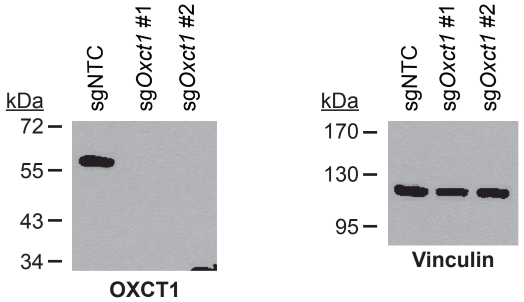

ED Figure 3i

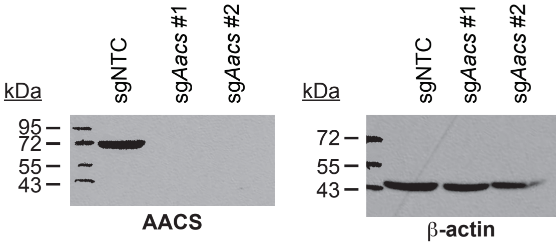

ED Figure 3m

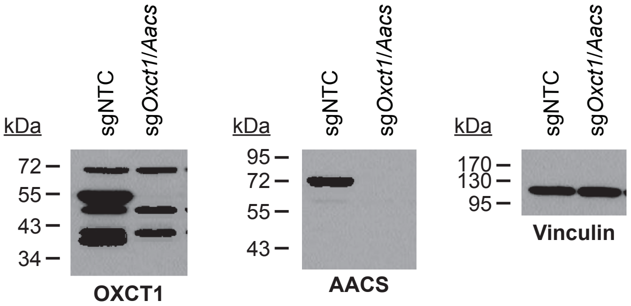

Supplement: Supplementary file 17 — Unprocessed western blots for Extended Data Fig. 3. [file 42255_2025_1366_MOESM17_ESM.pdf]
