# Supplementary material for: An alternative route for β-hydroxybutyrate metabolism supports cytosolic acetyl-CoA synthesis in cancer cells
Source: Nat Metab. 2025 Sep 8;7(10):2033–44. doi: 10.1038/s42255-025-01366-y (PMC12552118; doi:10.1038/s42255-025-01366-y)

Source Data Extended Data Figure 7. Uncropped western blot images.

ED Figure 7a

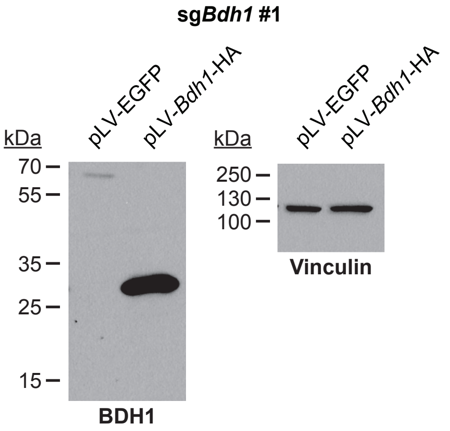

ED Figure 7d

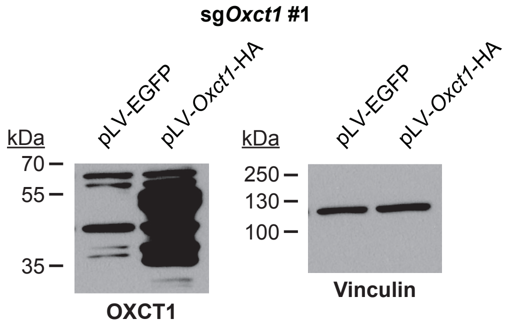

ED Figure 7g

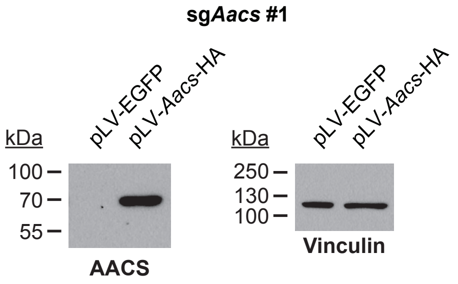

Supplement: Supplementary file 18 — Unprocessed western blots for Extended Data Fig. 7. [file 42255_2025_1366_MOESM18_ESM.pdf]
